# Aminal-Linked Porous Piperazine Covalent Organic Polymers for Gold Sequestration from E‑Waste with Exceptional Performance Metrics

**DOI:** 10.1021/acsami.5c25235

**Published:** 2026-05-14

**Authors:** Sushil Kumar, Mahira Bashri, Najat Maher Aldaqqa, Safa Gaber, Blaž Belec, Matthew A. Addicoat, Dinesh Shetty

**Affiliations:** † Department of Chemistry, Khalifa University of Science & Technology, Abu Dhabi 127788, United Arab Emirates; ‡ Center for Catalysis & Separation (CeCaS), Khalifa University of Science & Technology, Abu Dhabi 127788, United Arab Emirates; § Materials Research Laboratory, University of Nova Gorica, Vipavska 11c, Ajdovščina SI-5270, Slovenia; ∥ School of Science and Technology, Nottingham Trent University, Nottingham NG11 8NS, United Kingdom

**Keywords:** covalent organic polymers, e-waste, gold recovery, reusability, sustainability

## Abstract

Adsorption is a highly efficient, scalable, and economically viable strategy for gold recovery from electronic waste, yet its practical implementation depends critically on the development of adsorbents with exceptional selectivity and capacity. Here, we report the molecular design, synthesis, and comprehensive characterization of two aminal-linked covalent organic polymers (COPs), DSK-1 and DSK-2, incorporating intrinsic metal-chelating functionalities. Among these materials, DSK-1 exhibits an extraordinary gold adsorption capacity of 5560 mg g^–1^, as evaluated by a pseudo-second-order kinetic model—representing the highest capacity reported for COP-based adsorbents to date. Moreover, DSK-1 displays remarkable selectivity toward Au­(III) ions in complex electronic-waste leachates containing 12 competing metal ions, demonstrating its robustness under realistic extraction conditions. Collectively, these results underscore the potential of rationally engineered COPs as next-generation adsorbents for precious-metal recovery and highlight their promise as sustainable, high-performance materials for e-waste remediation.

## Graphical abstract

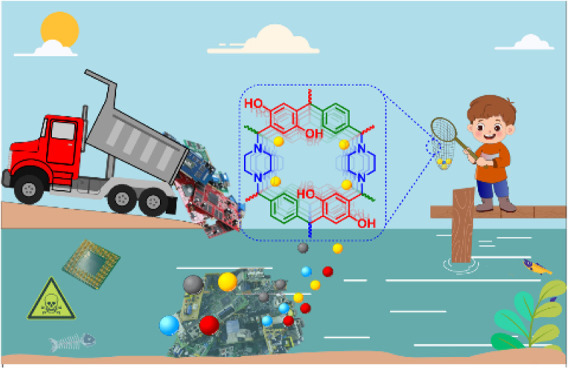

## Introduction

Recovery of precious resources from waste is a critical strategy for achieving sustainable development.^1,2^ Gold recovery from electronic waste (e-waste) has gained significant attention in recent years due to its economic and environmental implications. The global e-waste generation is projected to exceed 74 million metric tons by 2030, underscoring the urgent need for sustainable waste management protocols with minimal carbon footprints.^3^ Harvesting gold from e-waste not only mitigates the environmental damage caused by traditional mining practices—such as ecosystem degradation and high energy consumption—but also promotes resource conservation, economic growth, and transition to an avenue of circular economy.

Currently, several techniques, including electrolysis,^4^ oxidation,^5^ precipitation,^6^ adsorption,^7^ etc., have been explored for the recovery of gold from e-waste. Among these, adsorption stands out because of its cost-effectiveness, scalability, and energy efficiency. Conventional adsorbents, such as activated carbon, zeolites, and metal oxides, have been widely used but often suffer from limited selectivity and adsorption capacity in complex e-waste leachates.^8^ The lack of tailored functional sites restricts their ability to discriminate between competing metal ions, thereby reducing the efficiency in real-world applications. To overcome these limitations, advanced adsorbents with high surface area and engineered binding sites are essential.

Covalent organic frameworks (COFs) have emerged as a promising class of adsorbents because of their tunable porosity, chemical stability, and functional diversity.^9–11^ Recently, we have shown the importance of N_amine_ and N_bipyridine_ atoms within benzoxazine-core-based material to improve gold adsorption efficiency.^12^ These findings clearly highlight the importance of molecular design in enhancing the adsorption performance and selectivity. Covalent organic frameworks (COFs) are crystalline materials characterized by long-range order and periodic porous architectures, which endow them with predictable pore geometries, high surface areas, and unique physicochemical properties, such as efficient charge transport. In contrast, covalent organic polymers (COPs) generally form amorphous, noncrystalline networks.^13,14^ Although COPs retain key advantages—chemical robustness, permanent porosity, and structural tunability—the absence of long-range periodicity often results in broad pore-size distributions and materials designed primarily with processability in mind. Multicomponent reactions offer an effective strategy for precision engineering within covalently bonded networks, enabling the direct incorporation of functional binding sites into the polymer backbone through a single-step synthetic process. This approach also facilitates the formation of accessible active sites that can effectively target Au­(III) ions.

In this study, we explore the incorporation of piperazine units into COPs as a rational design strategy for the recovery of gold from electronic waste. Each piperazine moiety contains two tertiary nitrogen atoms bearing lone pairs that serve as strong Lewis bases capable of coordinating with Au­(III) species, consistent with hard–soft-acid–base (HSAB) principles. Such intrinsic binding functionality is expected to enhance the affinity and selectivity of the resulting COPs toward gold ions under complex leaching conditions.^15,16^ The real e-waste solutions are typically acidic in nature, in which gold ions often exist as [AuCl_4_]^−^ form. Under acidic pH, the piperazine can undergo protonation to form a piperazinium cation carrying a positive charge and thereby will facilitate a strong ion-pair interaction between the piperazinium moiety and [AuCl_4_]^−^ species. Moreover, we have incorporated the hydroxyl (−OH) functionality as an additional interaction site within the network backbone to further improve the ion-adsorption efficiency of the prepared COPs.^17,18^ In short, the incorporation of piperazine and hydroxyl functionality into the network structure not only increases the density of adsorption sites but also provides sites that facilitate adsorption and the reduction of Au^3+^ to Au^0^.

Herein, we have realized the above-mentioned molecular-level designing by synthesizing two aminal-linked COPs—DSK-1 (with backbone hydroxyl groups) and DSK-2 ^19^ (without backbone hydroxyl groups)—via a multicomponent Petasis reaction.^20^ The rationally designed DSK-1 with designated ion binding sites resulted in a high gold adsorption efficiency value of 5560.31 mg g^–1^, compared with the efficiency for DSK-2 (1622 mg g^–1^). To the best of our knowledge, the gold adsorption efficiency value observed for DSK-1 is the highest reported value for any porous network material with >99% gold uptake from real e-waste leachates in the presence of 12 competing metal ions. These results underscore the critical role of metal-selective binding sites within solid sorbents for maximizing gold recovery efficiency by offering a sustainable solution for e-waste valorization.

## Experimental Section

### Materials and Characterization Techniques

The starting materials, i.e., 2,5-dihydroxyterephthaldehyde (Dha), piperazine (Pz), 1,4-phenylenediboronic acid (Ba), and 1,4-phenylenediamine (Pa), were purchased from Aldrich and used as received without further purification. All solvents used for carrying out the synthesis were dried and distilled. A Rigaku Smart Lab II with a Cu Kα (λ = 1.5405 Å) radiation source operating at 40 kV and 40 mA was used for powder X-ray diffraction measurement. FT-IR spectra were taken on a Bruker Optics ALPHA-E spectrometer with a universal Zn–Se attenuated total reflection (ATR) accessory in the 630–4000 cm^–1^ region or using a Diamond ATR (Golden Gate) with a 16 scan rate and 4 cm^–1^ resolution. SEM analysis of COF samples was performed using a FEI Nova NanoSEM 650, which features an electron column with semi-in-lens detectors and an in-lens Schottky field-emission gun, delivering ultrahigh resolution over a wide range of probe current (1 pA to more than 200 nA). The images were recorded at a flow rate of 3.5 keV. TEM images were recorded using an FEI Tecnai TEM at 200 kV. XPS analysis was performed using a Supra+ instrument (Kratos, Manchester, UK) equipped with an Al Kα source and a monochromator with a takeoff angle of 90°. The charge neutralizer was turned on during the measurements, and data processing was performed by using ESCApe 1.5 software (Kratos). Porosity analyses were performed using the Anton Paar Autosorb iQ combined physisorption and chemisorption instrument. 20–30 mg of COF samples were used for each analysis. Details of sample preparations for characterizations are provided in Section S1 of Supporting Information.

## Methodology

### Synthesis of Aminal-Linked COPs

An oven-dried thick-walled pressure tube was charged with dialdehyde, i.e., 2,5-dihydroxyterephthaldehyde (Dha) or terephthaldehyde (Ta; 50 mg, 0.3 mol), 1,4-diboronic acid (Dba;0.49 mg, 0.3 mol), and piperazine (Pz; 26 mg, 0.3 mol) dissolved in 3 mL of ethanol and water (1:1, v/v). The solution was sonicated for 15 min. To this was added 50 μL of 6 M acetic acid, followed by sonication for 15 min. After sonication, the tube was heated at 110 °C for 24 h to afford a dark brown powder. Purification was performed by using a Soxhlet apparatus with THF and methanol for washing. The obtained material was then dried in an oven at 90 °C for 12 h prior to characterization.

### Gold Adsorption Studies

#### Adsorption Isotherm

20 mL of AuCl_3_ solutions at concentrations ranging from 1 to 1000 mg L^–1^ were allowed to adsorb with 2 mg of adsorbent until equilibrium was reached. The samples were filtered using 0.45 μm Nylon syringe filters, and changes in metal concentrations were monitored by ICP-MS. Adsorption isotherm fittings were done using the equations S2–S4 and Section S3 of Supporting Information.

#### Adsorption Kinetics

Into 100 mL of AuCl_3_ solution (50 mg L^–1^), 10 mg of adsorbent was added. Several samplings were collected over time from 0 to 48 h and filtered using 0.45 μm Nylon syringe filters. The filtrate samples were analyzed using ICP-MS to obtain residual metal concentration. Parameters of adsorption kinetics were determined using equations S5–S9 and Section S3 of Supporting Information.

#### Regeneration Experiment

First, 50 mL of a 20 mg L^–1^ AuCl_3_ solution was mixed with 10 mg of DSK-1 and stirred for 48 h. The gold-adsorbed polymer was thoroughly washed and dried. For desorption, the gold-adsorbed polymer was added to the stripping solution (50 mL of 0.5 M thiourea and 0.5 M HCl) and stirred for 24 h. The experiment was repeated for up to 5 cycles. Residual metal concentrations were analyzed after each adsorption, and respective uptake efficiencies were calculated. Additionally, the regeneration efficiency was monitored by calculating the desorption efficiency.

#### E-Waste Analysis

The e-waste leaching solution was prepared by soaking the central processing unit (CPU) in 50 mL of aqua regia for 24 h. The leaching solution was further diluted to pH 2. Twenty mL of e-waste leaching solution was treated with 2 mg of polymer under constant stirring for 48 h. The samples were filtered using 0.45 μm Nylon syringe filters, and changes in metal concentrations were monitored by ICP-MS.

## Results and Discussion

DSK-1 and DSK-2 COPs were prepared using a multicomponent Petasis reaction via a solvothermal approach (Figure 1a). Following a general synthesis method, Pz, Dba, and Dha or Ta were suspended in an ethanol and water (1:1 v/v) mixture in a Pyrex glass tube. After sonication for 15 min, the mixture was heated to 120 °C for 72 h. Both COPs were isolated as light brown solid samples (Section S2) and purified by the Soxhlet method using THF and methanol prior to characterization studies. The FTIR spectrum (Figure S2) of DSK-1 displayed an −OH stretch at 3372 cm^–1^, a C–N stretch at 1290 cm^–1^, and an —C=C— stretch at 1603 cm^–1^. However, the presence of a weak shoulder at 1644 cm^–1^ along with a broad −OH stretch suggests the existence of free aldehyde and a trace of carboxylic functionality in the structure. On the other hand, DSK-2 exhibits a C=O stretching band at 1686 cm^–1^ (Figure S3). The —C=C— and −C–N– stretching bands observed at 1604 and 1275 cm^–1^, respectively, confirm the formation of an aminal-linked network. As shown in Figure 1b, SSNMR spectra of DSK-1 and DSK-2 reveal distinct carbon environments within the material. For DSK-1, the carbon bearing the hydroxyl group resonates at approximately 152 ppm. The methylene carbons of the piperazine (Pz) unit appear as two distinct signals at 19 and 51 ppm, consistent with its chair conformation within the polymer backbone. In addition, the methylene carbon adjacent to the aminal linkage resonates at around 79 ppm. The aromatic carbons are observed across the expected range of 100–160 ppm. In the case of DSK-2, the signals appear broader—likely due to its higher degree of amorphous character relative to DSK-1—yet the major resonances corresponding to aromatic and aliphatic carbons remain clearly assignable.

**Figure 1:**
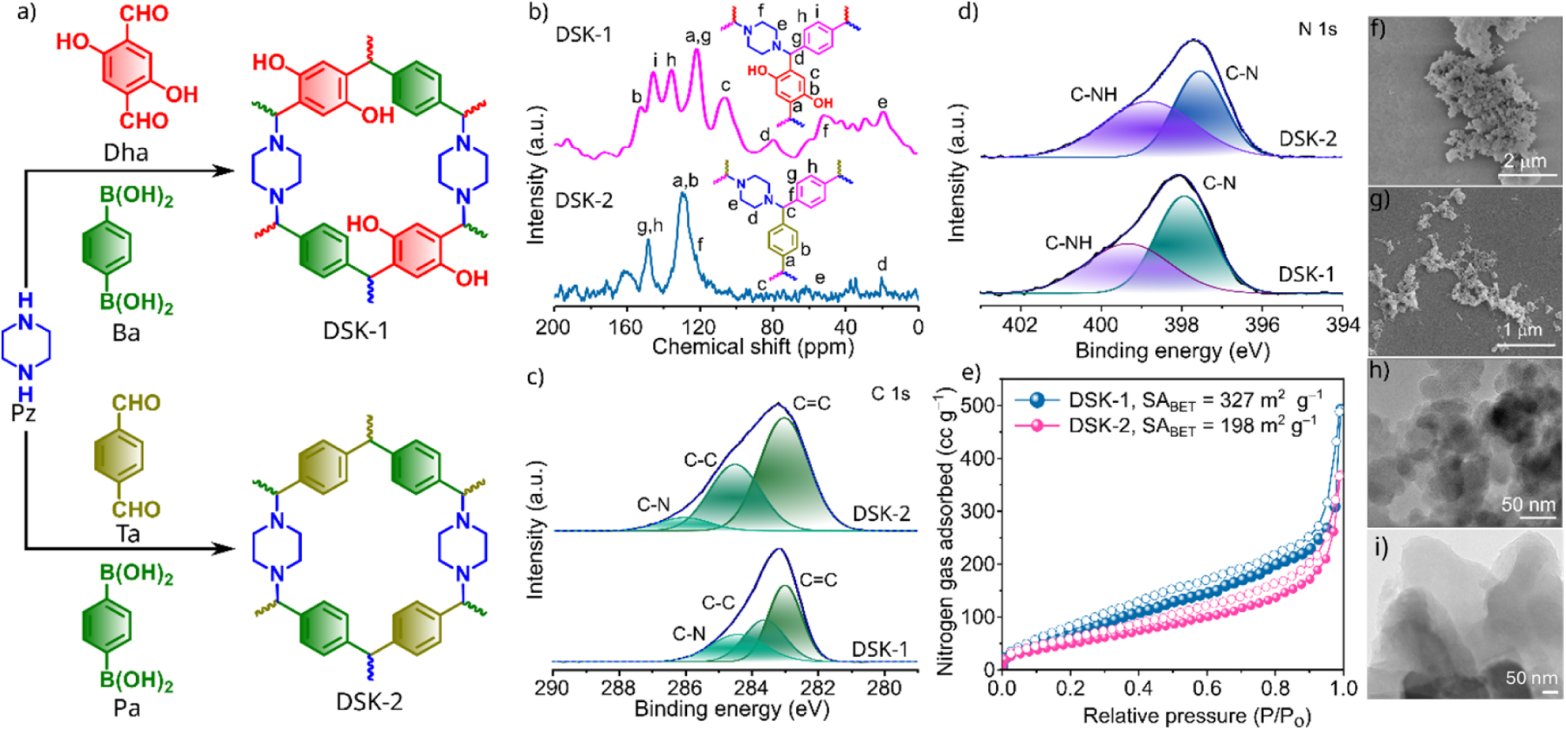
(a) Covalent organic networks synthesized via the Petasis reaction method. b) Solid-state ^13^C NMR spectra. Deconvoluted c) C 1s and d) N 1s XPS profiles. e) BET isotherm, SEM images of f) DSK-1 and g) DSK-2. TEM images of h) DSK-1 and i) DSK-2.

X-ray photoelectron spectroscopy (XPS) analysis further verified the extent of the reaction between the monomers and confirmed the successful formation of the aminal-linked framework (Figures S4–S5). The survey spectrum of DSK-1 displayed three primary peaks at binding energies of 283, 398, and 531 eV, corresponding to the C 1s, N 1s, and O 1s signals, respectively (Figure S4). Deconvolution of the high-resolution C 1s spectrum revealed two major peaks at 282.98 and 283.60 eV, which can be attributed to C=C and C–C functionalities (Figure 1c). A third peak at 284.40 eV was assigned to overlapping contributions from C–O and C–N bonds, consistent with the formation of the aminal linkage. The high-resolution N 1s spectrum exhibited a dominant peak at 397.92 eV, characteristic of C–N bonds originating from the incorporated piperazine units, thereby confirming their integration into the polymer backbone (Figure 1c). A secondary signal at 399.32 eV was attributed to adventitious nitrogen species or partially reacted piperazine groups. Deconvolution of the O 1s region showed two peaks at 531.11 and 533.01 eV, corresponding to phenolic C–O groups and trace adventitious aldehyde species (Figure S4). For DSK-2, the XPS survey revealed signals assigned to C 1s, N 1s, and O 1s (Figure S5). The C 1s spectrum showed peaks at 283.02, 284.51, and 286.04 eV, associated with C=C, C–C, and C–N functionalities (Figure 1c). The N 1s region displayed two peaks at 397.54 and 398.51 eV, corresponding to the C–N bond and residual or adventitious piperazine (C–NH) groups (Figure 1d). Notably, both the C 1s and N 1s signals in DSK-1 appear to be shifted to slightly lower binding energies relative to DSK-2. This shift is attributed to increased bond polarization arising from the hydroxyl groups embedded within the DSK-1 network, which modify the local electronic environment around the carbon and nitrogen atoms.

The powder X-ray diffraction (PXRD) studies of our synthesized aminal-linked COPs were performed between 2θ = 2.5° and 50° (Figure S6). This yields the amorphous polymeric networks.

To determine the porous nature of the prepared COPs, we recorded the nitrogen gas sorption profiles of DSK-1 and DSK-2. As illustrated in Figure 1e, both COPs exhibit a characteristic type II isotherm according to the IUPAC classification. Analysis of the isotherms facilitated the determination of Brunauer–Emmett–Teller (BET) surface areas, yielding 327 m^2^ g^–1^ (DSK-1) and 198 m^2^ g^–1^ (DSK-2). The pore size distribution curves show a uniform pore distribution in the range of 1.6–8 Å with average sizes of 2.8 Å (for DSK-1) and 4.8 Å (for DSK-2), as shown in Figures S7–S8. The morphological characteristics of the synthesized COPs were examined through scanning electron microscopy (SEM) and transmission electron microscopy (TEM). SEM micrographs of both COPs revealed sheet-like morphology, a typical feature for powder materials (Figure 1f and g). This observation was corroborated by TEM images, which further highlighted the sheet-like structure in both samples (Figure 1h and i). Notably, the sheet morphology of DSK-2 was more pronounced, likely due to its semicrystalline nature, as indicated by the sharp diffraction peaks in its PXRD pattern. It is important to distinguish the amorphous polymeric networks synthesized in this work from crystalline COFs, which are defined by long-range periodic order in two or three dimensions, typically evidenced by sharp Bragg reflections in PXRD and often accompanied by high, well-defined porosity (e.g., BET surface areas exceeding 1000 m^2^ g^–1^). In contrast, the materials reported here, DSK-1 and DSK-2, display featureless PXRD patterns (Figure S6), confirming the absence of extended crystallinity. Their measured BET surface areas—327 and 98 m^2^ g^–1^, respectively—are significantly lower than those characteristic of highly porous COFs. These textural and structural characteristics are more consistent with those of CONs or amorphous porous COPs, which possess covalent connectivity and short-range order but lack the periodic lattice required to classify them as COFs. Accordingly, although synthesized using a related dynamic covalent chemistry, DSK-1 and DSK-2 are most appropriately described as amorphous network polymers or COPs.

The electron-rich nitrogen atoms within the piperazine linker act as strong Lewis bases. According to the HSAB theory, soft donor sites exhibit high affinity and selectivity for soft metal ions, facilitating effective coordination and ion-exchange processes. Incorporating piperazine units directly into the polymer backbone, therefore, introduces a high density of well-defined binding sites, substantially enhancing the material’s metal ion adsorption capacity.^1,6^ Motivated by these characteristics, we evaluated the adsorption performance of the synthesized COPs toward Au­(III) ions in aqueous media. Adsorption studies were carried out by agitating aqueous AuCl_3_ solutions (300 ppm) with a known mass of each COP at 150 rpm and ambient temperature until equilibrium was achieved. Gold uptake was quantified by inductively coupled plasma mass spectrometry (ICP-MS) analysis of the filtered solutions. Detailed experimental procedures are provided in Section S3 of the Supporting Information.

The adsorption kinetics of Au­(III) ions, present predominantly as [AuCl_4_]^−^ species under acidic conditions, were evaluated by monitoring the uptake efficiency over time at pH 2 (Figure 2a). Both DSK-1 and DSK-2 rapidly reduced the aqueous gold concentration, achieving nearly 50% removal within the first 30 min. This rapid initial uptake can be attributed to the high density of accessible protonated piperazine units, which readily coordinate with Au­(III) ions and drive early-stage adsorption. DSK-1 demonstrated markedly higher adsorption performance, removing over 92% of Au­(III) within 2 h and achieving quantitative uptake (>99%) after 48 h. In contrast, DSK-2 reached equilibrium at a lower capacity, removing 82.3% of the initial Au­(III) concentration. The superior performance of DSK-1 is attributed to the presence of additional hydroxyl groups within its framework, which can engage in hydrogen-bonding interactions with the [AuCl_4_]^−^ species. These cooperative interactions complement the coordination provided by the piperazine nitrogen atoms and collectively enhance gold uptake. Kinetic modeling using the pseudo-first-order and pseudo-second-order models (Figures S15–S16) revealed that adsorption onto both materials was best described by the pseudo-second-order model, with excellent correlation coefficients (R^2^ = 0.99). For DSK-1, the pseudo-second-order rate constant was determined to be 2.01 × 10^–4^ g mg^–1^ min^–1^, while DSK-2 displayed a rate constant of 2.54 × 10^–4^ g mg^–1^ min^–1^. The strong agreement with the pseudo-second-order model indicates that chemisorption—likely involving electron sharing or transfer between the Au­(III) species and the nitrogen-containing binding sites—is the dominant adsorption mechanism for both COPs.

**Figure 2:**
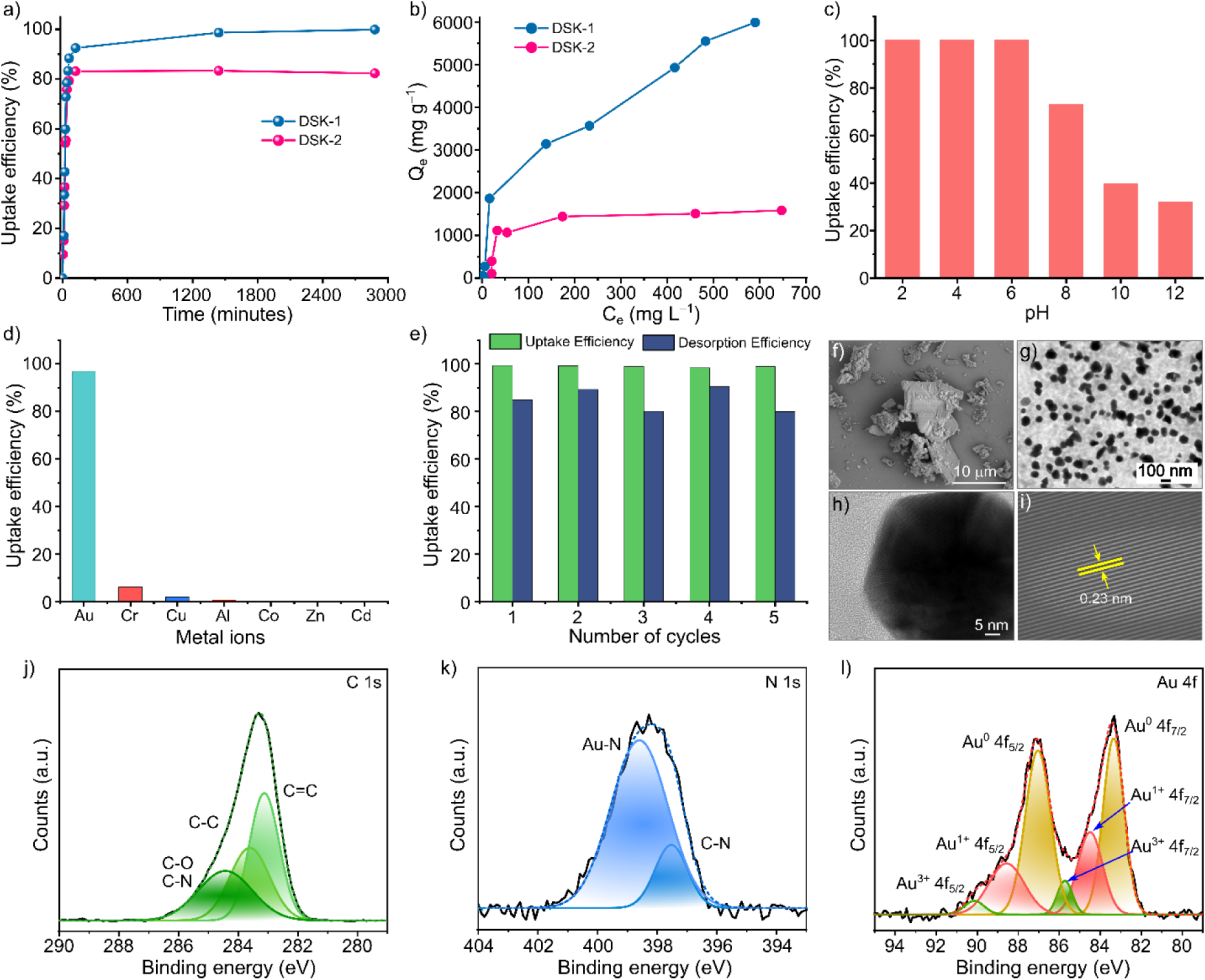
Adsorption kinetics of a) Au@DSK-1 and Au@DSK-2, b) adsorption isotherm of Au@DSK-1 and Au@DSK-2, c) gold adsorption efficiency of DSK-1 at different pH, d) selective gold uptake efficiency of DSK-1 from a mixture of different metal ions present in equimolar concentration, e) recyclability performance of DSK-1 in gold adsorption, f) SEM image of Au@DSK-1, g) TEM image of Au@DSK-1, h) single gold nanoparticle, i) lattice fringes, XPS profile: j) C 1s, k) N 1s, and l) Au 4f.

To further elucidate the adsorption mechanism and surface interactions between Au­(III) species and the COP networks, the equilibrium adsorption data were fitted to both the Langmuir and Freundlich isotherm models (Figures S17–S18). The isotherms for Au^3+^—present predominantly as [AuCl_4_]^−^ ions prior to their partial reduction to gold nanoparticles—were found to align closely with the Langmuir model. This indicates that gold uptake proceeds primarily through monolayer adsorption on a finite number of energetically uniform binding sites within the COPs. This Langmuir-type behavior is consistent with the nitrogen-rich microenvironment of the polymers; under acidic conditions, the piperazine units become protonated to form piperazinium (−NH^+^) sites. These positively charged amine centers act as well-defined and spatially discrete binding sites for the anionic [AuCl_4_]^−^ complexes. Once these sites are saturated, further adsorption is limited, thereby preventing multilayer accumulation—an outcome that contrasts with the assumptions of the Freundlich model, which is applicable to heterogeneous surfaces and multilayer sorption. The maximum adsorption capacities (Q_m_) calculated from the Langmuir fits were 5560 mg g^–1^ for DSK-1 and 1622 mg g^–1^ for DSK-2 (Figure 2b), revealing a substantial difference in their gold-uptake performance. The exceptionally high Q_m_ value of DSK-1 highlights the synergistic contribution of its structural features: beyond the piperazine units, the additional hydroxyl groups facilitate hydrogen bonding interactions with [AuCl_4_]^−^, thereby strengthening binding and enhancing overall adsorption capacity.^17,18^ , In contrast, DSK-2 lacks these auxiliary functional sites and, therefore, relies solely on piperazine-mediated coordination, leading to lower gold uptake.

The stability and efficiency of an adsorbent across diverse chemical environments are critical factors for its practical applicability. Accordingly, the chemical stability of DSK-1 was evaluated over a broad pH range (1–12). The FTIR spectra (Figure S15) and PXRD patterns (Figure S16) showed no detectable changes after exposure, confirming that the structural integrity of DSK-1 is well retained under both acidic and alkaline conditions. Because gold recovery processes typically operate in strongly acidic environments, we next assessed the pH-dependent adsorption performance of DSK-1 using solutions adjusted to pH 2–12 (Figure 2c). DSK-1 exhibited exceptional uptake efficiency (>99.9%) within the pH range of 2–6. However, adsorption efficiency progressively declined above pH 8, with pronounced loss of performance at pH 12. To rationalize this behavior, we measured zeta potentials and examined the chemical environment around the piperazine nitrogen atoms (N_piperazine_) and hydroxyl oxygen atoms (O_hydroxyl_), both of which interact with [AuCl_4_]^−^. Under acidic conditions (pH 2–6), N_piperazine_ becomes protonated (−N → −NH^+^), and the O_hydroxyl_ groups remain partially protonated or form stabilizing hydrogen bonding interactions.^1,8^ These protonation events impart a localized positive charge to the polymer framework, enabling strong electrostatic attraction toward anionic [AuCl_4_]^−^ complexes. This trend is reflected in the positive zeta potential values observed under acidic conditions (Table S4). The combination of electrostatic attraction, hydrogen bonding, and coordination creates a highly polarized microenvironment that significantly enhances the affinity of DSK-1 for Au­(III), surpassing what would be expected from single-site interactions alone. At pH values above 7, the solution becomes increasingly alkaline, causing the deprotonation of hydroxyl groups and the formation of alkoxide (−O^–^) species. This introduces localized negative charge on the polymer backbone, consistent with the decrease and eventual inversion of zeta potential values under basic conditions. Simultaneously, [AuCl_4_]^−^ undergoes hydrolytic transformation into less electrophilic species such as [Au­(OH)_3_Cl]^−^ and [Au­(OH)_4_]^−^.^1,2^ These hydrolyzed complexes exhibit weaker interactions with deprotonated binding sites. The combination of reduced electrophilicity and electrostatic repulsion between negatively charged gold hydroxyl species and negatively charged polymer sites leads to diminished gold uptake under alkaline conditions. In summary, the high adsorption efficiency of DSK-1 in acidic media arises from protonation-induced positive surface charge and synergistic N/O binding interactions, whereas deprotonation and gold species hydrolysis under alkaline conditions lead to substantial performance loss.

High selectivity toward gold in the presence of competing metal ions is essential for practical e-waste recycling, where Au­(III) is typically present at extremely low concentrations relative to those of other metals. The presence of competing ions can reduce adsorption efficiency and compromise the viability of the adsorbent for real-world applications. Common interfering ions in e-waste leachates include Cu^2+^, Ni^2+^, Co^2+^, and Cd^2+^, among others. To evaluate the selectivity, DSK-1 (chosen as the representative material due to its superior performance) was tested for Au­(III) adsorption in the presence of equimolar competing metal ions: Cu^2+^, Cr^3+^, Al^3+^, Zn^2+^, Co^2+^, and Cd^2+^ (Figures 2d and S10). Remarkably, DSK-1 demonstrated a gold uptake efficiency of 97%, far exceeding the adsorption efficiency of the other ions. Minimal uptake was observed for Cr^3+^ (6.2%), Cu^2+^ (2.03%), Al^3+^ (0.40%), and Co^2+^ (0.0042%), while Zn^2+^ and Cd^2+^ showed no detectable adsorption. These results confirm that competing ions exert a negligible influence on [AuCl_4_]^−^ adsorption, and DSK-1 maintains strong selectivity for gold under multicomponent conditions. This behavior can be rationalized using HSAB (Hard–Soft Acid–Base) theory. Under the acidic conditions used, most competing ions remain as cations, leading to electrostatic repulsion from the positively charged, protonated piperazinium sites on DSK-1. In contrast, gold exists as the soft anionic complex [AuCl_4_]^−^, which interacts strongly with the protonated piperazine units and hydroxyl groups via electrostatic attraction and complementary coordination. This charge complementarity gives DSK-1 a substantial selectivity advantage over cation-binding systems. To assess reusability—a critical parameter for scalable metal recovery—the adsorption performance of DSK-1 was evaluated over five consecutive adsorption/desorption cycles (Figure 2e). Gold desorption was achieved using a thiourea solution, followed by a HCl wash to remove residual thiourea and regenerate the adsorbent. PXRD and FTIR analyses of regenerated materials confirmed the preservation of structural integrity and the complete removal of thiourea (Figures S21–S22). DSK-1 maintained an excellent gold uptake efficiency of >99.34% even after five cycles, demonstrating strong recyclability. Desorption efficiencies were calculated by comparing the recovered gold concentration in the eluent to the amount adsorbed in each preceding cycle. The efficiencies remained consistently high—85.2%, 89.6%, 80.5%, 91.0%, and 80.3% for cycles 1–5, respectively. The minor decline (less than 15–18% over all cycles) aligns with the slight decrease in adsorption capacity shown in Figure 2e and indicates that DSK-1 possesses robust regeneration capability. Overall, the material’s outstanding selectivity, excellent recyclability, and minimal structural degradation highlight its strong potential for practical gold recovery from complex e-waste environments.

The DSK-1 obtained after gold adsorption studies is further termed as Au@DSK-1. While comparing the FT-IR spectrum of Au@DSK-1 with pristine DSK-1, a shift in the C–N stretch to a lower wavenumber and broadness of the C–OH stretch suggest the interaction of gold with the N_piperazine_ and O_hydroxyl_ atoms within the COPs (Figure S19). While FT-IR spectroscopy revealed the initial binding sites for gold adsorption on the DSK-1 skeleton through characteristic C–N peak shifts, PXRD studies can provide complementary evidence by confirming this Au–N interaction. In this aspect, the PXRD pattern of Au@DSK-1 for pristine and after adsorption of Au^3+^ ions was recorded. According to our observation, characteristic diffraction peaks corresponding to metallic gold (Au^0^) nanoparticles were observed (Figure S20). Specifically, prominent peaks at 2θ = 38.2° and 44.4° were observed, attributable to the diffraction from (111) and (200) crystallographic planes of Au^0^, respectively.^21,22^ This observation provides compelling evidence that Au^3+^ ions undergo reduction to Au^0^ nanoparticles upon interaction with the functional groups, i.e., N_piperazine_ and O_hydroxyl_ present in the skeleton of DSK-1. In other words, the functionally active sites present in DSK-1 not only adsorb Au^3+^ ions but also facilitate their transformation into the Au^0^ state. To gain further insight into Au binding in these CONs from a molecular perspective, we used density functional theory (DFT) to calculate the binding energies for Au(0) and AuCl_4_^–^ in DSK-1 and DSK-2. The computed binding energies are −70.4 and −118.0 kJ mol^–1^ for Au(0) and AuCl_4_^–^, respectively, in DSK-1, and −137.9 and −122.2 kJ mol^–1^ in DSK-2 (Table S7). These values indicate strong stabilization of both Au(0) and AuCl_4_^–^ within the pores, with particularly favorable binding of Au(0) in DSK-2. The optimized structures show that Au(0) binds primarily to hydroxy groups covering the pores of the CONs.

To substantiate this observation and gain deeper insight into the mechanism, detailed morphological investigations were conducted using SEM and TEM. These complementary techniques offer insight into the structural and compositional transformations during gold adsorption, reinforcing the proposed reduction mechanism of Au^3+^ to Au^0^. Analysis of SEM images of Au@DSK-1 revealed a notable morphological change, with the formation of a plate-like structure larger than that of the pristine samples, as shown in Figure 2f. This structural transformation suggests that gold incorporation influences the material’s surface characteristics, potentially through the deposition and growth of metallic particles.

In further morphological characterizations, the TEM images of Au@DSK-1 distinctly show the Au^0^ NP embedded within the DSK-1 matrix (Figure 2g). This micrograph highlights the hexagonal morphology of the particles due to the templating or stabilizing effect of the CON structure during the reduction and nucleation of the gold. To probe the crystalline nature of these particles, high-resolution TEM (HRTEM) imaging was employed. Focused on single nanoparticles (Figure 2h), the HRTEM micrograph showcased well-defined lattice fringes, indicating the high crystallinity of gold nanoparticles. The measured *d*-spacing value of ∼0.23 nm (for Au@DSK-1) aligns closely with the (111) lattice plane of metallic gold, corroborating the PXRD data and reinforcing the conclusion that Au^3+^ is reduced to Au^0^ (Figure 2i). Quantitative analysis of the NP size distribution, derived from TEM observations, indicated an average gold particle size of 74.42 ± 15.91 nm (Figure S21). To determine the distribution and elemental composition of gold within the matrix, energy-dispersive X-ray (EDX) mapping studies were performed, which demonstrated a uniform distribution of gold particles throughout the sample matrix (Figures S22–S25). This uniform distribution underscores the efficiency of DSK-1 as a platform for both capturing and reducing gold ions, ensuring that the resulting NPs are well integrated into the material rather than merely aggregated in specific regions. Based on XPS quantitative analysis, we estimate that approximately 62.91% of the adsorbed gold is in the reduced Au^0^ state, while 6.02% remains as Au^3+^, and the rest is present in the Au^1+^ form.

Although both DSK-1 and DSK-2 remove approximately 50% of Au­(III) within the first 30 min, the overall adsorption kinetics reach equilibrium over a longer period (up to 48 h). This extended time scale can be attributed to the combined influence of the materials’ amorphous, sheetlike morphology and the multistep adsorption–reduction mechanism proposed for gold uptake. In contrast to rigid, highly porous adsorbents where physisorption and rapid intraparticle diffusion dominate, the gold-capture process in these COPs involves not only the initial coordination of Au^3+^ species to piperazine and hydroxyl functionalities but also their subsequent in situ reduction to Au^0^ nanoparticles. Evidence of this reduction pathway is supported by PXRD, XPS, and TEM analyses. The formation and growth of Au^0^ particles within the polymer matrix are inherently slower than surface adsorption alone, thereby contributing to the overall kinetic profile. Additionally, the layered and somewhat compact morphology observed in SEM and TEM images (Figure 1f–i) likely imposes greater mass transfer resistance than that in highly mesoporous or fibrous adsorbents, further influencing the rate of equilibrium attainment. Despite these modest kinetic constraints, the materials—particularly DSK-1—exhibit exceptional performance, achieving an ultrahigh maximum capacity (5560 mg g^–1^) and outstanding selectivity in complex e-waste leachates. The ability to directly convert Au^3+^ into recoverable metallic gold within the polymer framework provides an added functional and economic advantage that offsets the slower adsorption kinetics.

To further validate the gold adsorption mechanism, we conducted a comprehensive XPS survey. The XPS full survey profile of Au@DSK-1 revealed five prominent peaks corresponding to Au 4f, Cl 2p, C 1s, N 1s, and O 1s (Figure S26). For in-depth analysis, the high-resolution XPS spectra of the constituent elements were deconvoluted. The high-resolution C 1s spectrum displayed three distinct signals at 283.11, 283.60, and 284.42 eV, which were attributed to the binding energies of C=C, C–C, and C–O/C–N bonds, respectively (Figure 2j). Similarly, the N 1s spectrum revealed two peaks: the first at 397.54 eV, corresponding to the C–N bond within the aminal linkage, and the second at 398.61 eV, indicative of Au–N interactions (Figure 2k). Deconvolution of the O 1s spectrum uncovered two signals: the peaks at 531.17 and 533.04 eV. A small shift of approximately 0.03–0.06 eV in the peaks observed in the O 1s profile for Au@DSK-1 compared to pristine DSK-1 suggests an involvement of the −OH group in a weak hydrogen bonding interaction with [AuCl_4_]^−^ ions (Figure S26d). The high-resolution Au 4f spectrum presented six distinct peaks: signals at 83.33 and 87.01 eV corresponded to Au^0^ 4f_7/2_ and Au^0^ 4f_5/2_ states, 84.47 and 88.53 eV were associated with Au^1+^ 4f_7/2_ and Au^1+^ 4f_5/2_ states, and peaks at 85.70 and 90.10 eV were attributed to Au^3+^ 4f_7/2_ and Au^3+^ 4f_5/2_ states (Figure 2l). Notably, the predominance of Au^0^ peaks suggests that a significant proportion of adsorbed Au^3+^ ions were reduced to Au^1+^ and Au^0^ states. In short, the above findings, PXRD and morphological studies, displayed a detailed picture of the adsorption-reduction process facilitated by the synergistic effect of heteroatoms present within the DSK-1 during the gold recovery process.

To achieve selective gold recovery from complex e-waste, a leaching solution was prepared by dissolving a discarded central processing unit (CPU) in aqua regia. The detailed methodology for preparing this solution is elaborated in Section S3 of Supporting Information. The resulting acidic leach solution, characterized by a highly acidic pH of ∼ 1, contained a diverse array of metal ions, reflecting the intricate composition of e-waste. Among these metal ions, Cu^2+^ ions were present in a high concentration of approximately 142 times that of Au^3+^, while Ni^2+^ and Al^3+^ were present at approximately 12.8 and 3 times the concentration of Au^3+^, respectively, as illustrated in Figure 3a. Other metal ions, including Ag^+^, Pb^2+^, Ca^2+^, V^3+^, Cr^3+^, Mn^3+^, Fe^3+^, Co^2+^, and Zn^2+^ were detected at concentrations comparable to that of Au^3+^, underscoring the challenge of selective adsorption of gold from such a complex solution (Figure 3a). Despite the overwhelming presence of competing metal ions, particularly the dominant Cu^2+^, Ni^2+^, and Al^3+^, the DSK-1 demonstrated remarkable selectivity and an uptake efficiency of 99.2% in gold adsorption, as shown in Figure 3b. In addition, the DSK-1 regenerated after the adsorption process was characterized via FTIR and PXRD studies, suggesting the structure of the DSK-1 is well maintained and fully suitable for the next round of the gold adsorption process (Figures S27–S30).

**Figure 3:**
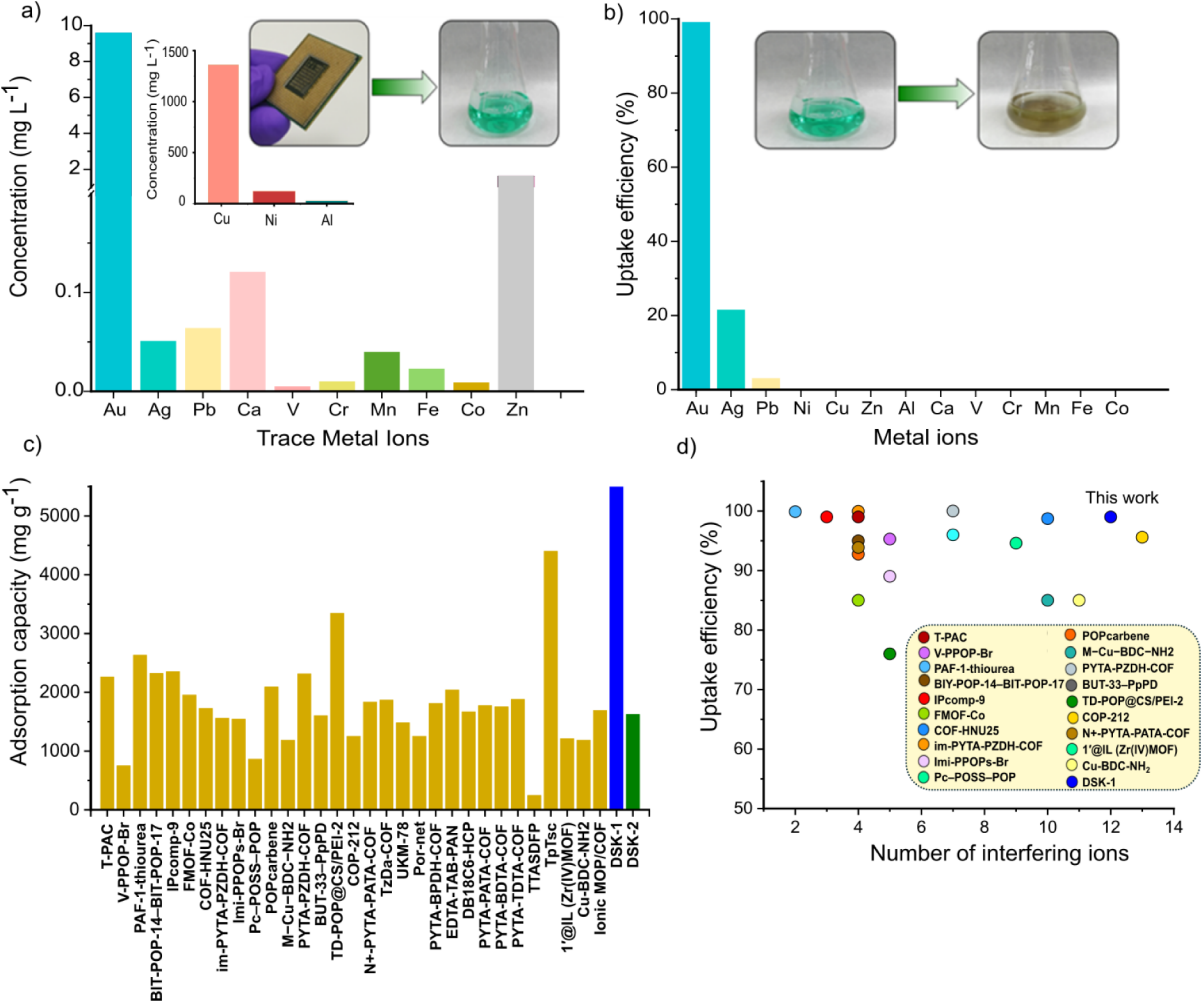
(a) Concentration and type of different metal ions present in the acidic leachate prepared by dissolving CPU in aqua regia, b) selective uptake efficiency of DSK-1 from real e-waste, c) comparison of adsorption capacity of different materials for gold ions reported in literature for gold recovery, and d) uptake efficiency of reported literature examples, including DSK-1, in the presence of a number of interfering ions.

To emphasize the remarkable gold adsorption performance of DSK-1, we conducted a comparative analysis of its maximum adsorption capacity (Q_m_) for Au^3+^ ions against a range of reported porous polymeric materials. Notably, DSK-1 demonstrated the highest Q_m_ value, emphasizing its potential as a high-performance material in this domain (Figure 3c and Table S6). In addition, we compared the selectivity performance of literature examples by plotting their uptake efficiency against the number of interfering ions ranging from 2 to 13. As shown in Figure 3d, DSK-1 exhibits an uptake efficiency of >99% in the presence of 12 interfering ions, making it competitive with several literature examples and suggesting excellent selectivity. Looking ahead, we believe that designing advanced structures enriched with strategically positioned heteroatoms could simultaneously preserve the chemical robustness and stability of the framework. Furthermore, the use of adsorptive measures is expected to enhance the selectivity and effectiveness of gold recovery in future applications, paving the way for more efficient and sustainable solutions in precious metal recycling technologies.

## Conclusions

In summary, we have demonstrated the successful synthesis of piperazine-containing COPs via a three-component Petasis reaction and evaluated their performance for high-capacity gold recovery from electronic waste leachates. Among the materials developed, DSK-1 exhibited an exceptional Q_m_ of 5560 mg g^–1^ and achieved >99% adsorption efficiency under optimized conditions. Moreover, DSK-1 showed outstanding selectivity for Au­(III) even in the presence of multiple competing metal ions commonly found in e-waste, highlighting its robustness and potential for practical recovery processes. The heteroatom-rich framework—featuring both nitrogen and oxygen functionalities—played a pivotal role not only in the strong adsorption of Au­(III) species but also in their subsequent in situ reduction to metallic gold. Evidence from PXRD, XPS, and morphological analyses confirms that this reduction is facilitated by the unique chemical environment within the COP structure. Collectively, these findings provide valuable insight into the rational design of functionalized polymers for targeted metal capture.

## Supplementary Material



## Notes

Experimental details, FT-IR data, XPS data, fractional atomic coordinates, gold adsorption kinetic and characterization data, SEM, TEM, and EDX data (PDF)
