# Supplementary material for: Aminal-Linked Porous Piperazine Covalent Organic Polymers for Gold Sequestration from E‑Waste with Exceptional Performance Metrics
Source: ACS Appl Mater Interfaces. 2026 May 14;18(20):29045–53. doi: 10.1021/acsami.5c25235 (PMC13220223; doi:10.1021/acsami.5c25235)
Supplement: Supplementary file 1 [file am5c25235_si_001.pdf]

Supporting Information

Aminal-linked Porous Piperazine Covalent Organic Polymers for Gold Sequestration from E-Waste with Exceptional Performance Metrics

Sushil Kumar,[a b ‡] *Mahira Bashri,*[a ‡] *Najat Maher Aldaqqa,*[a,b] *Safa Gaber,*[b] *Blaž Belec,*[c] *Matthew A. Addicoat,*[d] *and Dinesh Shetty*[*a b]

[a]Department of Chemistry, Khalifa University of Science & Technology, Abu Dhabi, United Arab Emirates. E-mail: dinesh.shetty@ku.ac.ae

[b]Center for Catalysis & Separation (CeCAS), Khalifa University of Science & Technology, Abu Dhabi, United Arab Emirates

[c]Materials Research Laboratory, University of Nova Gorica, Vipavska 11c, Slovenia

[d]School of Science and Technology, Nottingham Trent University, Nottingham, United Kingdom.

‡ Authors contributed equally to this work

Section S1. Materials and methods

The starting material i.e. 2,5-dihydroxyterephthaldehyde (Dha), piperazine (Pz), 1,4-phenylenediboronic acid (Ba), and 1,4-phenylenediamine (Pa) were purchased from Aldrich and used as received without further purification. All solvent used for carrying out the synthesis were dried and distilled.

General instrumentation methods and sample preparation

Powder X-ray Diffraction (PXRD): Rigaku Smart Lab II with Cu Kα (λ = 1.5405 Å) radiation source operating at 40 kV and 40 mA was used for Powder X-ray diffraction measurement. The samples were analysed over the 2θ range of 2.5-50° with step size of 0.02° (divergent slit=1/16°).

Fourier Transform Infrared (FT-IR): FT-IR spectra were taken on Bruker Optics ALPHA-E spectrometer with a universal Zn-Se attenuated total reflection (ATR) accessory in the 630-4000 cm^{-1} region or using a Diamond ATR (Golden Gate) with 16 scan rate and 4 cm^{-1} resolution.

Scanning Electron Microscopy (SEM): SEM analysis of COP samples were analyzed using FEI Nova NanoSEM 650 consists of an electron column with semi-in-lens detectors and an in-lens Schottky field emission gun to deliver ultra-high resolution with a wide range of probe current (1 pA to more than 200 nA). The images were recorded at a voltage of 3.5 keV. Samples for SEM analysis was prepared by drop-casting (~10 µL) the material dispersed in isopropyl alcohol over clean silicon substrate. The dried samples were coated with Pt (nano-sized film) using JEOL JEC-300FC Auto Fine prior to the analysis.

Transmission Electron Microscopy: TEM images were recorded using FEI Tecnai TEM 200 kV. Samples were prepared by drop casting powder dispersion in isopropyl alcohol over carbon coated copper grids TEM Window (TED PELLA, INC. 200 mesh).

X-ray Photoelectron Spectroscopy (XPS): XPS analysis was performed using Supra+ instrument (Kratos-Manchester, UK) equipped with an Al Kα source and a monochromator with a take-off angle 90°. The charge neutralizer was turned on during the measurements and data processing was performed using ESCApe 1.5 software (Kratos). The samples were placed over silicon wafer attached to carbon tape. The analysis area was 300×700 microns with pass energy of 20 eV and base pressure of main analysis chamber at 2×10^{-9} mbar. Binding energy scale correction was done based on C-C/C-H peak at 284.8 eV in the C 1s spectra.

Gas Adsorption: Porosity analyses were performed using the Anton Paar Autosorb iQ combined physisorption and chemisorption instrument. 20-30 mg of COP samples were used for each analysis. Prior to doing N_2 gas adsorption in liquid N_2 bath (77K) for collecting full isotherm, the samples were activated by keeping at 80 °C for 16 hours. Surface area was calculated using multipoint Brunauer-Emmet-Teller (BET) model, whereas pore size distribution was found using the non-local density functional theory (NLDFT).

BET theory is an extension of Langmuir theory, which is widely used to determine the adsorption of gas molecules forming a monolayer on a solid surface; monolayer formation is related to the physical adsorption of gas molecules on a solid surface. BET equation describes the relationship between the number of gas molecules adsorbed at a given relative pressure. Therefore, it serves as the basis for the measurement of the specific surface area of a material.

The BET equation is (Eq. S1)

$$\frac{1}{v[(p_o/p)-1]} = \frac{c-1}{v_m c}\left(\frac{p}{p_o}\right)+\frac{1}{v_m c}$$

where p and p_o are the equilibrium and saturation pressure of adsorbents at the adsorption temperature; v is the adsorbed gas quantity (for example, in volume units); and v_m is the adsorbed gas quantity on the monolayer; c is the BET constant.

Density Functional Theory (DFT) calculations:

Geometries of DSK-1 and DSK-2 COPs were constructed using AuToGraFS [10.1021/jp507643v] and optimized in monolayer, AA, slipAA and AB configurations using Density Functional Tight Binding (DFTB). To determine the binding energies of adsorbed gold species in the frameworks, DFT binding energies were calculated using BLYP-D3(BJ) / TZP in AMS2024.105. [SCM] Monolayer structures were cut from the calculated AA structures of DSK-1 and DSK-2 and capped. Au(0) and $AuCl_4^-$ were added to multiple positions within the pore and optimised with COP backbone atoms fixed.

[SCM] AMS 2024.105, SCM, Theoretical Chemistry, Vrije Universiteit, Amsterdam, The Netherlands, http://www.scm.com

Section S2. Synthesis procedure

General synthesis procedure. An oven dried thick walled pressure tube was charged with dialdehyde i.e. Dha or Ta (50 mg, 0.3 mol), Ba (0.49 mg, 0.3 mol), and Pz (26 mg, 0.3 mol) dissolved in 3 mL of ethanol and water (1 : 1, v/v). The solution was sonicated for 15 minutes. To this was added 50 µL of 6 M acetic acid followed by sonication for 15 minutes. After sonication, the tube was then subjected to heating at 110 °C for 24 h to afford dark brown powder. Purification was done by Soxhlet setup using THF and methanol for washing. Obtained material was then dried in an oven maintained at 90 °C for 12 h prior to characterization studies.

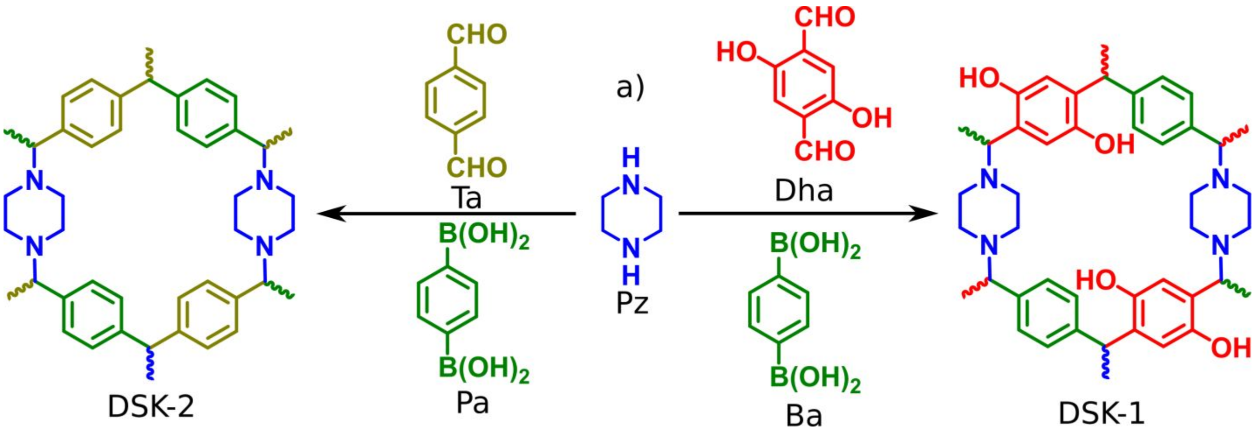

Figure S1. Synthesis of **DSK-1 and DSK-2**.

Section S3. Gold adsorption studies

Adsorption isotherm: 20 mL $AuCl_3$ solutions with concentrations ranging from 1 to 1000 mg L^{-1} was allowed to adsorb with 2 mg of adsorbent until reaches equilibrium. The samples were filtered using 0.45 µm Nylon syringe filters and change in metal concentrations were monitored *via* ICP-MS analysis.

Adsorption constant was calculated using the following equation.

$$Q_e = (C_0 - C_e) \times V/M \quad \text{(Eq. S2)}$$

Where C_0 and C_e are the initial and equilibrium concentrations, M is the mass of adsorbent in g, and V is the volume of solution in litre.

Linear Langmuir isotherm fitting was done using the following equation.

$$C_e/Q_e = (Q_m \times K_a)^{-1} + C_e/Q_m \quad \text{(Eq. S3)}$$

Linear Freundlich isotherm fitting was done using the following equation.

$$\ln Q_e = \ln K_f + 1/n \ln C_e \quad \text{(Eq. S4)}$$

Where Q_m is the maximum adsorption capacity, K_a, n and K_f are the adsorption constants.

Adsorption kinetics: Into a 100 mL, 50 mg L^{-1} $AuCl_3$ solution, 10 mg of adsorbent was added. Several samplings were collected with respect to time from 0 to 48 hours and filtered using 0.45 µm Nylon syringe filters. The filtrate samples were analysed using ICP-MS to obtain residual metal concentration.

Uptake efficiency was calculated using the following equation.

$$R_e\,(\%) = ((C_0 - C_e) \times 100)/C_e \quad \text{(Eq. S5)}$$

Pseudo first oder kinetic fittings were done using the following equation.

$$\ln(Q_e - Q_t) = \ln Q_e - k_1 t \quad \text{(Eq. S6)}$$

Pseudo second order fittings were done using the following equation.

$$t/Q_t = (k_2 Q_e^2)^{-1} + t/Q_e \quad \text{(Eq. S7)}$$

Where, t is the time (min) and k_1 and k_2 are the pseudo first and second order rate constants, Q_e and Q_t are the adsorption capacity (mg g^{-1}) at equilibrium and time t respectively.

Selectivity test: 100 mL of mixed metal ions solution with 50 mg L^{-1} each of Au^{3+}, Al^{3+}, Cr^{3+}, Co^{2+}, Cd^{2+}, Zn^{2+} and Cu^{2+} was freshly prepared. 10 mg adsorbent was added allowed to adsorb for 48 hours. Samplings were done at specific time intervals. The samples were filtered using 0.45 µm Nylon syringe filters and change in metal concentrations were monitored *via* ICP-MS analysis.

The distribution co-efficient (K_d) of each metal ion (in L /g) in real e-waste leach solution towards DSK-1 was calculated as follows;

$K_d = ((C_0-C_e) \times V)/M \times C_e = Q_e/C_e$ (Eq. S8)

***p*H study:** 2 mg each of adsorbents were added to 10 mL 50 mg L^{-1} $AuCl_3$ solutions prepared at varying *p*Hs (2,4,6,8,10 and 12) and stirred for 48 hours. The initial and final concentrations of the $AuCl_3$ solutions were analysed using ICP-MS after filtering the samples using 0.45 µm Nylon syringe filters. Subsequently, uptake efficiencies were calculated.

Regeneration experiment: First, 50 mL, 20 mg L^{-1} $AuCl_3$ solution were added with 10 mg of DSK-1 and allowed to stir for 48 hours. The gold adsorbed polymer was thoroughly washed and dried. For desorption, the gold adsorbed polymer was added into the stripping solution (mixed solution of 50 mL, 0.5 M Thiourea and 0.5 M HCl) and allowed stir for 24 hours. The regenerated polymer was washed with DI water for several times and reused for further adsorption cycles. The experiment was repeated for up to 5 cycles. Residual metal concentrations were analyzed after each adsorption and respective uptake efficiencies were calculated. Additionally, the regeneration efficiency was monitored by calculating the desorption efficiency.

Desorption efficiency (%)= $(C_d/C_0-C_e) \times 100$ (Eq. S9)

C_d, refers to the residual concentration of stripping solution after treating gold adsorbed DSK-1.

E-waste analysis: the e-waste leaching solution was prepared by soaking the central processing unit (CPU) in 50 mL aqua regia for 24 hours. The leaching solution was further diluted to a *p*H=2. 20 mL e-waste leaching solution was treated with 2 mg polymer with constant stirring for 48 hours. The samples were filtered using 0.45 µm Nylon syringe filters and change in metal concentrations were monitored *via* ICP-MS analysis.

Section S4. Characterization data

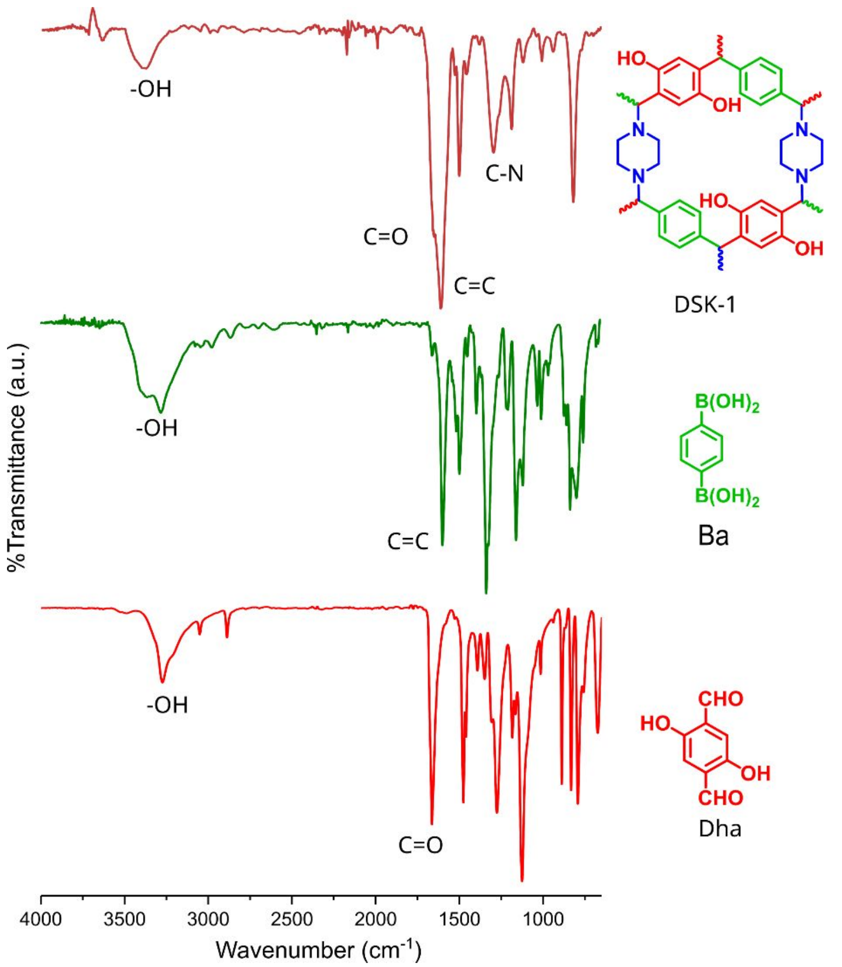

Figure S2. A comparison of FTIR spectra of DSK-1 with respective monomers.

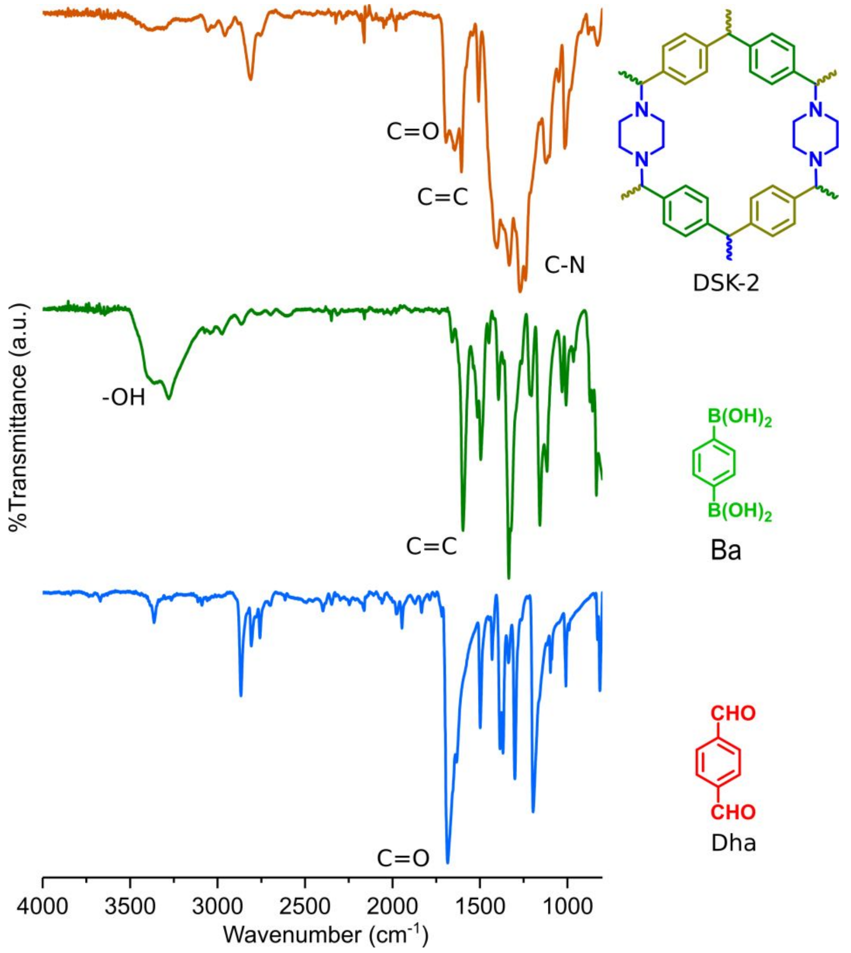

Figure S3. A comparison of FTIR spectra of DSK-2 with respective monomers.

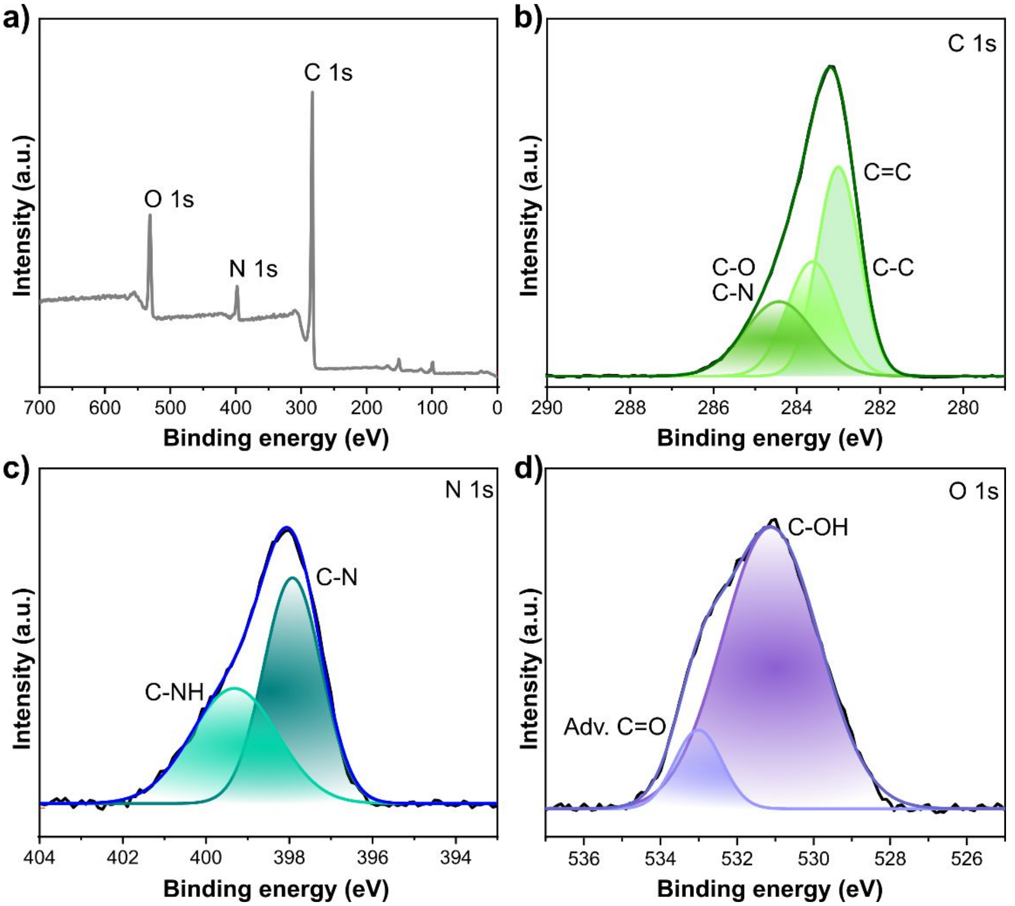

Figure S4. XPS profile of DSK-1: a) full survey, b) C 1s, c) N 1s, and d) O 1s.

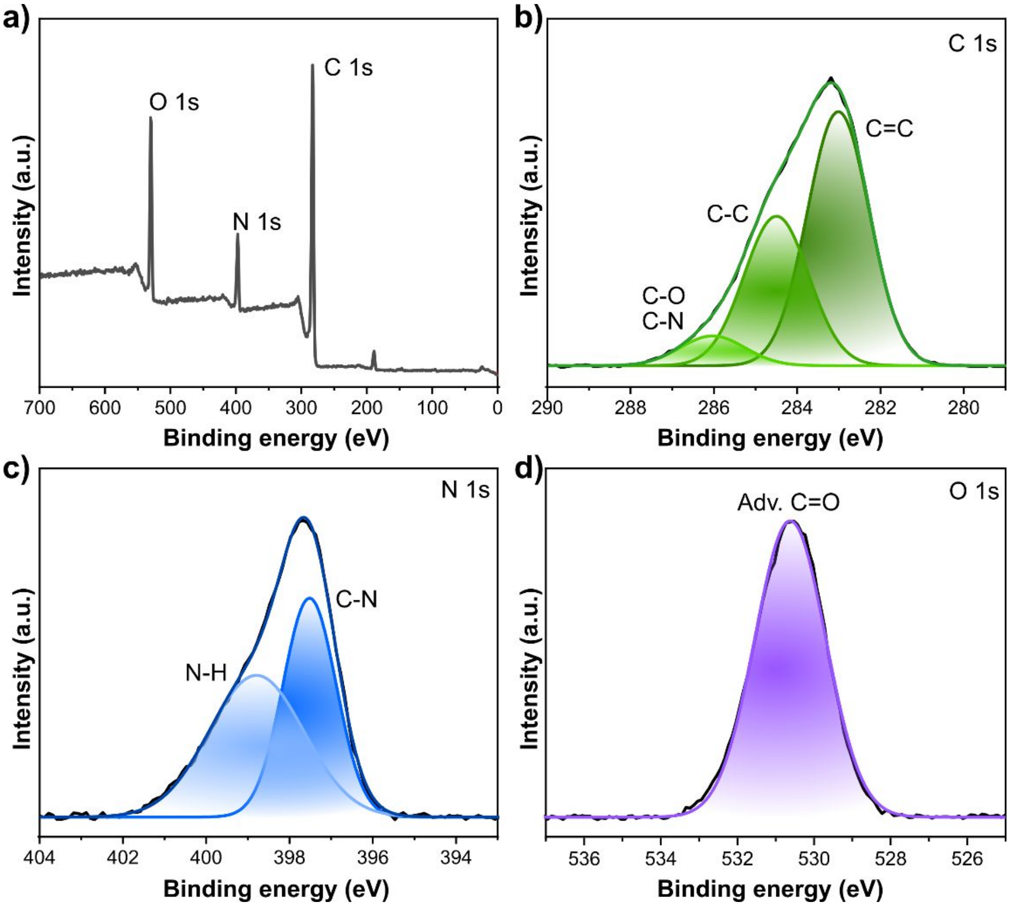

Figure S5. XPS profile of DSK-2: a) full survey, b) C 1s, c) N 1s, and d) O 1s.

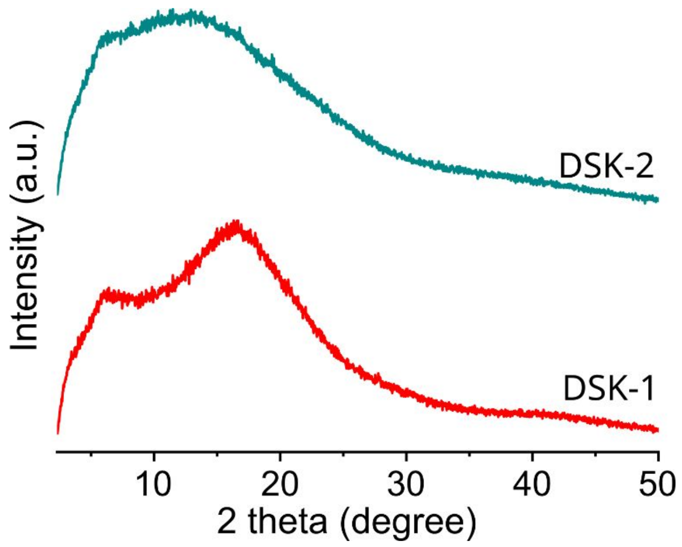

Figure S6. PXRD pattern of DSK-1 and DSK-2.

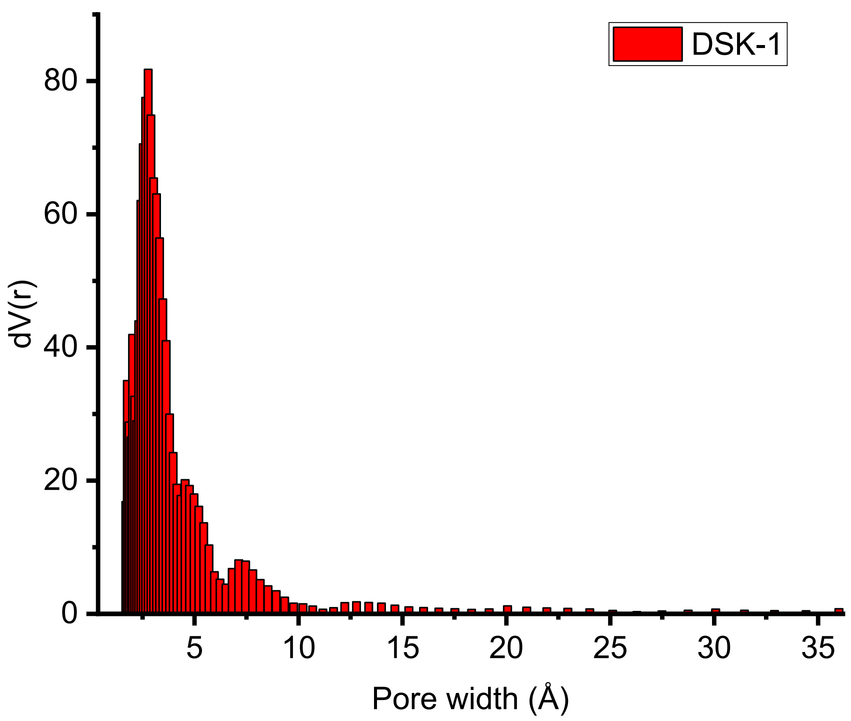

Figure S7. Pore size distribution curve for DSK-1.

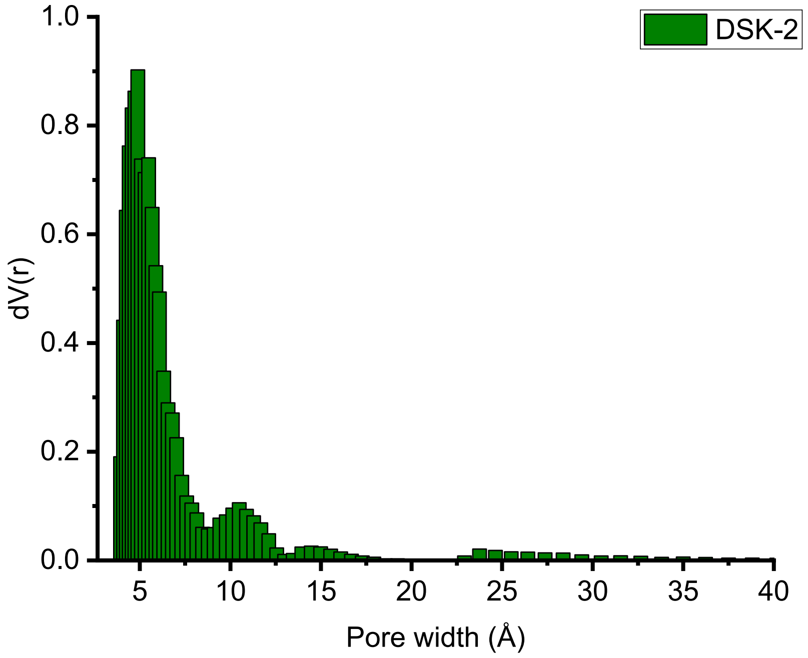

Figure S8. Pore size distribution curve for DSK-1.

Section S5. Gold adsorbed CONs and characterizations studies

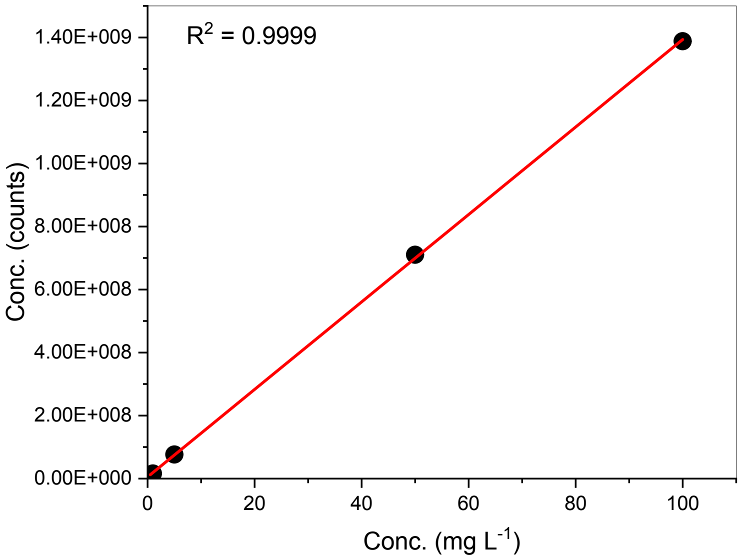

Figure S9. ICP calibration curve using different concentration of gold chloride solution.

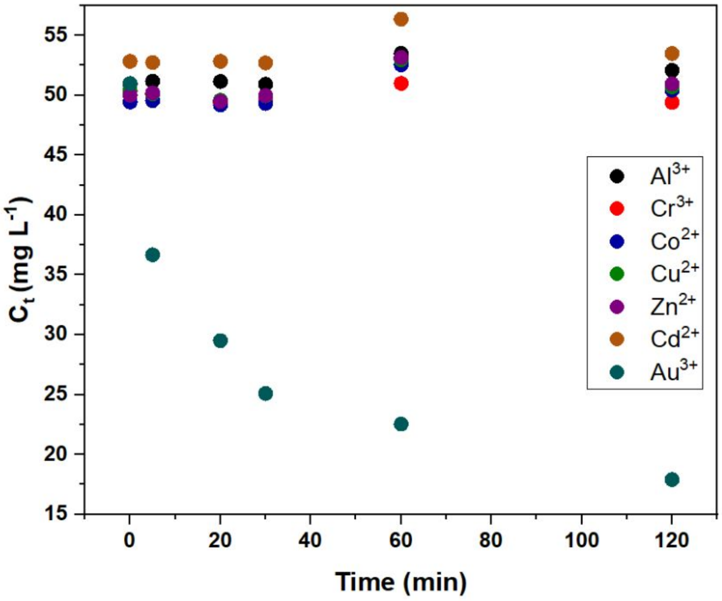

Figure S10. Selective adsorption of Au (III) towards DSK-1 with time compared to competing ions, when treated with laboratory simulated mixed metal ion solution.

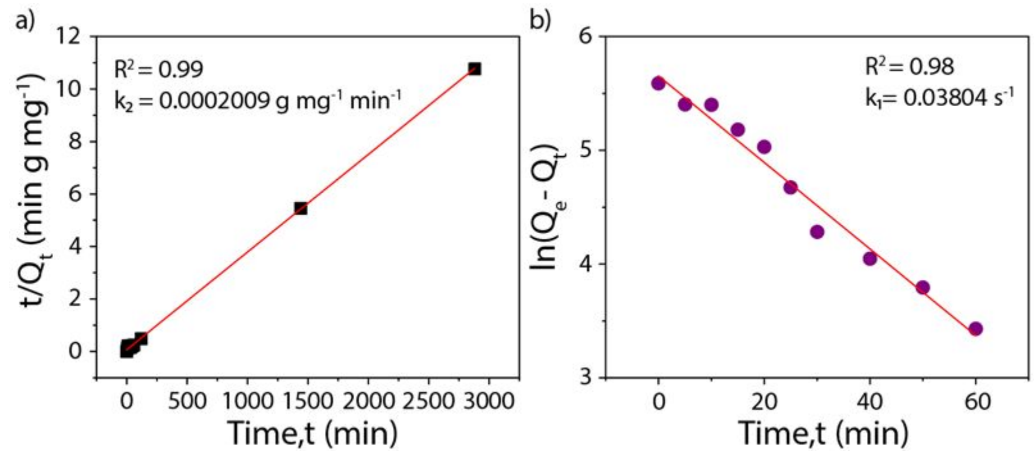

Figure S11. Kinetics fitting for DSK-1 in (a) pseudo second order and (b) pseudo first order model for gold adsorption**.**

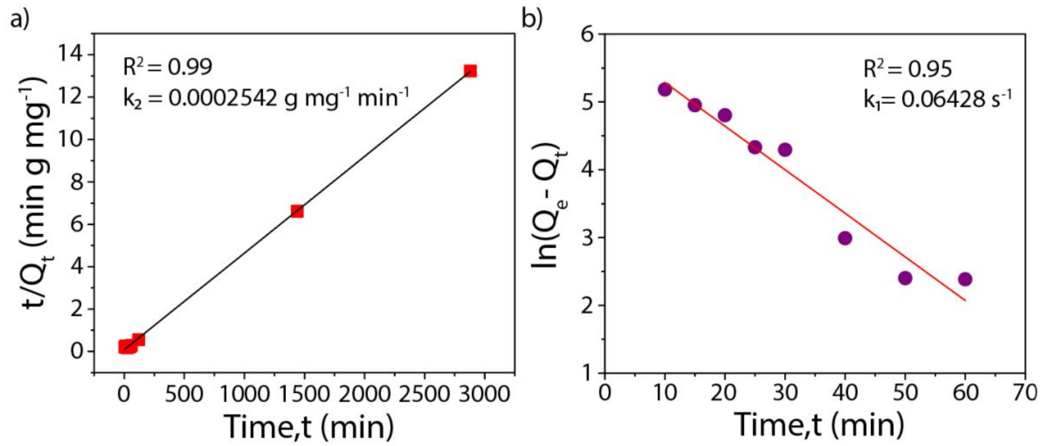

Figure S12. Kinetics fitting for DSK-2 in (a) pseudo second order and (b) pseudo first order model for gold adsorption**.**

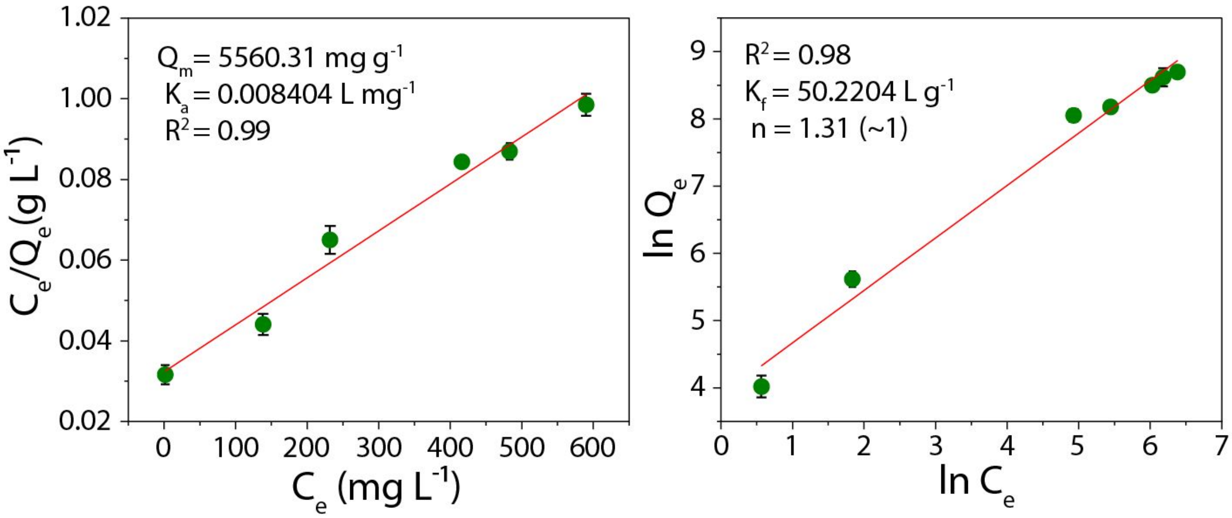

Figure S13. (a) Langmuir and (b) Freundlich isotherm fitting for DSK-1 in adsorption of gold ions.

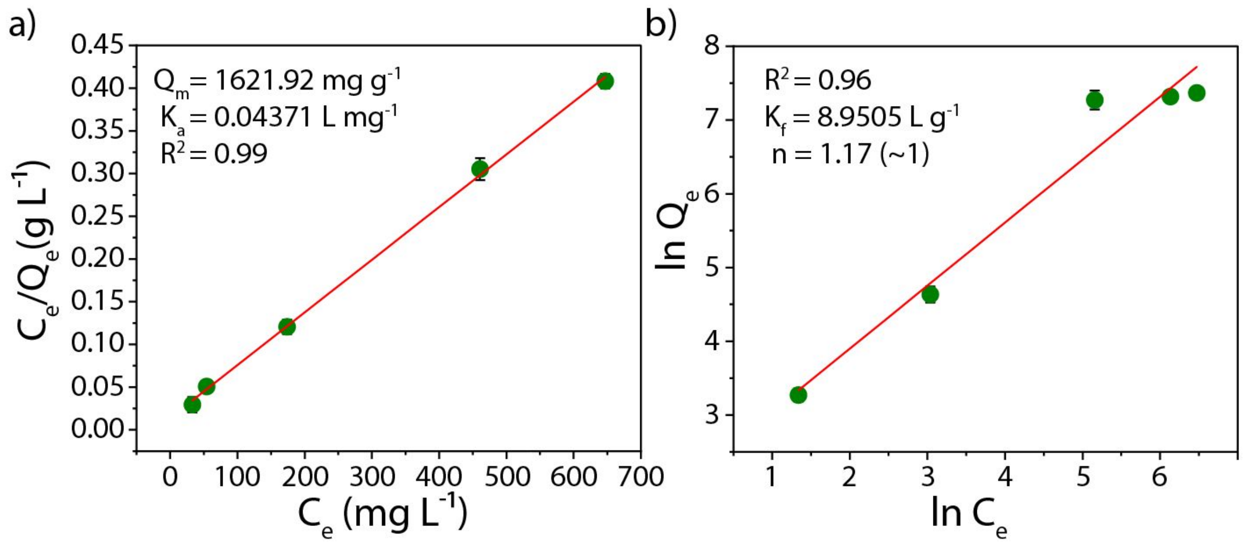

Figure S14. (a) Langmuir and (b) Freundlich isotherm fitting for DSK-2 in adsorption of gold ions.

Table S1. Calculated kinetics constants for DSK-1 and DSK-2.

**CON**	**Pseudo first order**		**Pseudo second order**	
	R^2	k_1 (s^{-1})	R^2	k_2 (g mg^{-1} min^{-1})
**DSK-1**	0.98	0.03804	0.99	0.0002009
**DSK-2**	0.95	0.06428	0.99	0.0002542

Table S2. Calculated adsorption constants for DSK-1 and DSK-2.

**CON**	**Freundlich Isotherm**			**Langmuir Isotherm**		
	R^2	K_f	n	R^2	K_a ($L\ mg^{-1}$)	Q_m ($mg\ g^{-1}$)
**DSK-1**	0.98	50.2204	1.31	0.99	0.008404	5560.31
**DSK-2**	0.96	8.9505	1.17	0.99	0.04371	1621.92

Table S 3. Distribution co-efficient of competing ions in e-waste w. r.t the DSK-1

Metal ions	K_d (L/g)
Au	60.83
Ag	0.23
Pb	0.03
Ni	0
Cu	0
Zn	0.01
Al	0.01
Ca	0
V	0

Cr	0.1
Mn	0
Fe	0
Co	0

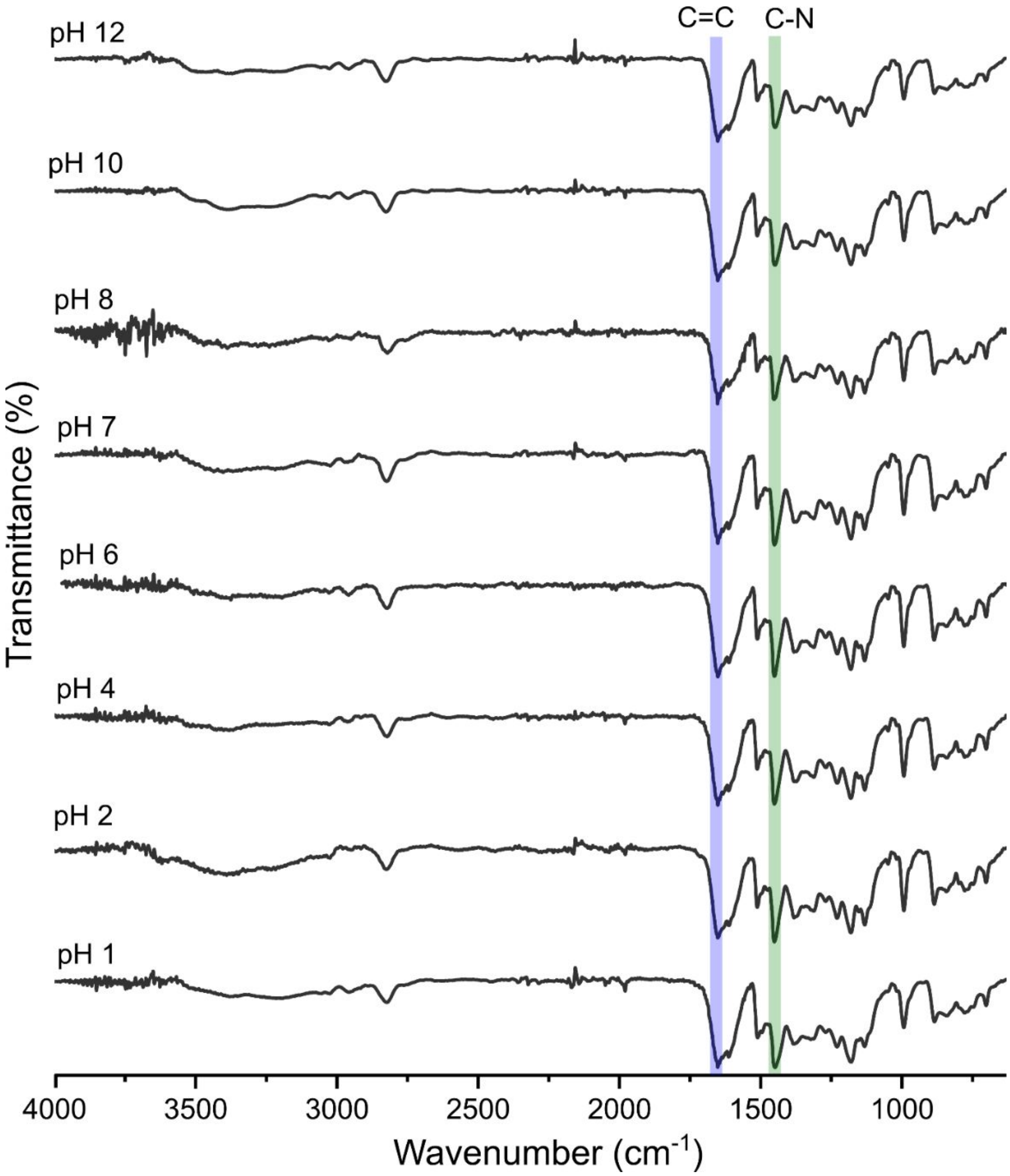

Figure S15. FTIR spectra of DSK-1 recorded after treatment at different pH.

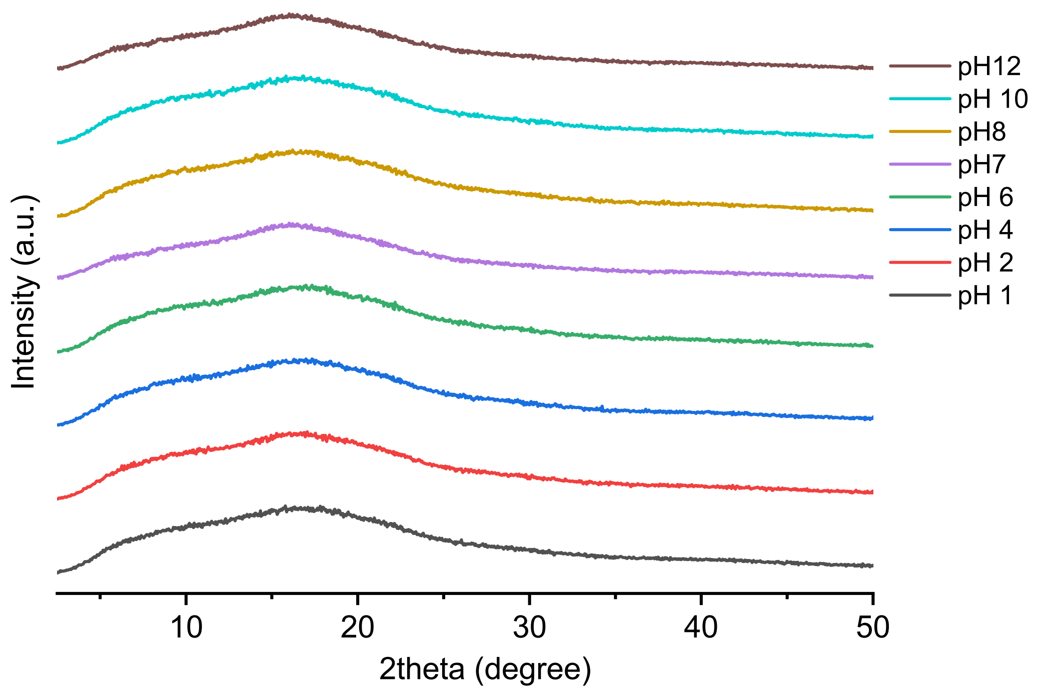

Figure S 16: PXRD patterns of DSK-1 treated at different pH.

Table S4. Zeta potential values measured for DSK-1 at different pH.

pH	Zeta potential (mV)	Average Zeta potential (mV)	Standard deviation
1	18.5	18.83333	0.757188
1	19.7		
1	18.3		
2	31.8	33	1.74356
2	32.2		
2	35		
4	65.4	65.96667	0.737111
4	66.8		
4	65.7		
6	64.5	66.93333	2.739221
6	69.9		
6	66.4		
7	90.6	85.3	4.986983
7	84.6		
7	80.7		
8	44.3	44.03333	1.02632
8	44.9		
8	42.9		
10	13.5	14.13333	0.568624
10	14.3		
10	14.6		
12	-60.5	-63.0667	2.289833
12	-63.8		
12	-64.9		

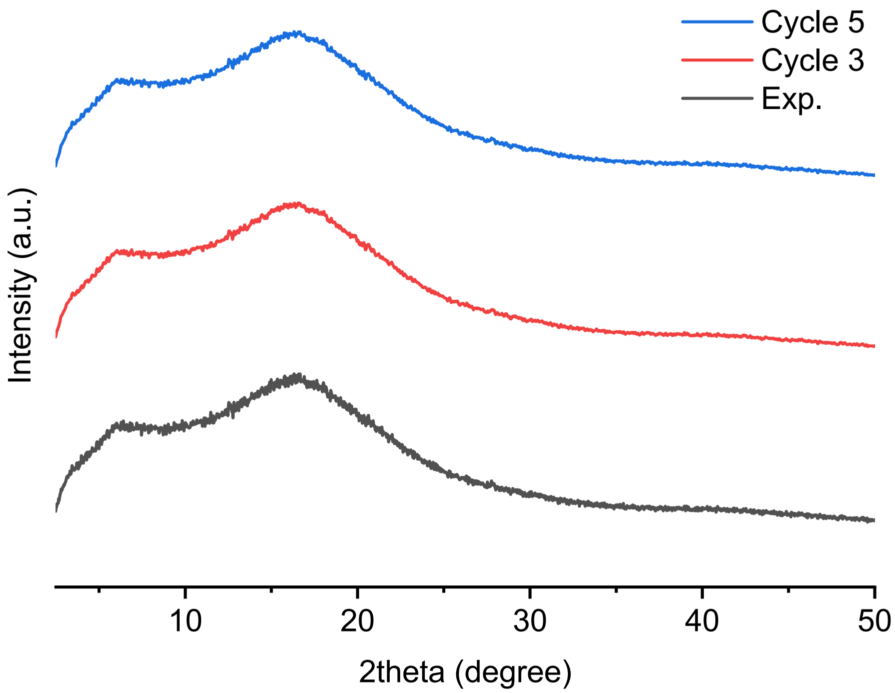

Figure S17. PXRD patterns of DSK-1 recovered after gold adsorption followed by the treatment with thiourea and hydrochloric acid.

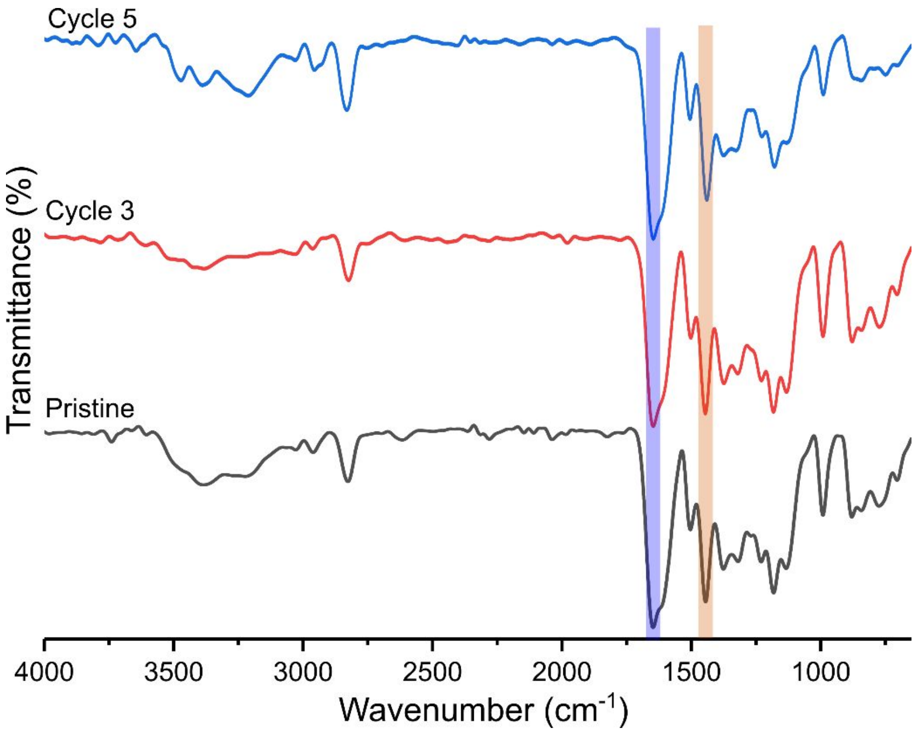

Figure S18. FTIR of DSK-1 recorded after treatment with gold solution following treatment with thiourea and hydrochloric acid.

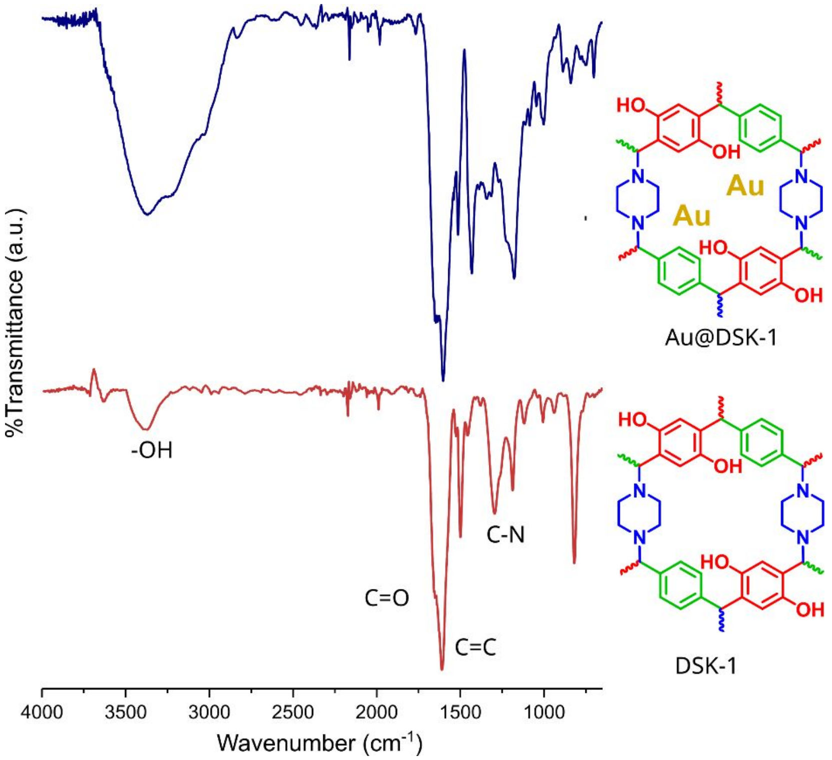

Figure S19. Comparison of FTIR spectra of Au@DSK-1 with pristine DSK-1 show shift of C-N stretch to higher wavenumber suggesting the interaction between $N_{piperazine}$ atoms and Au ions.

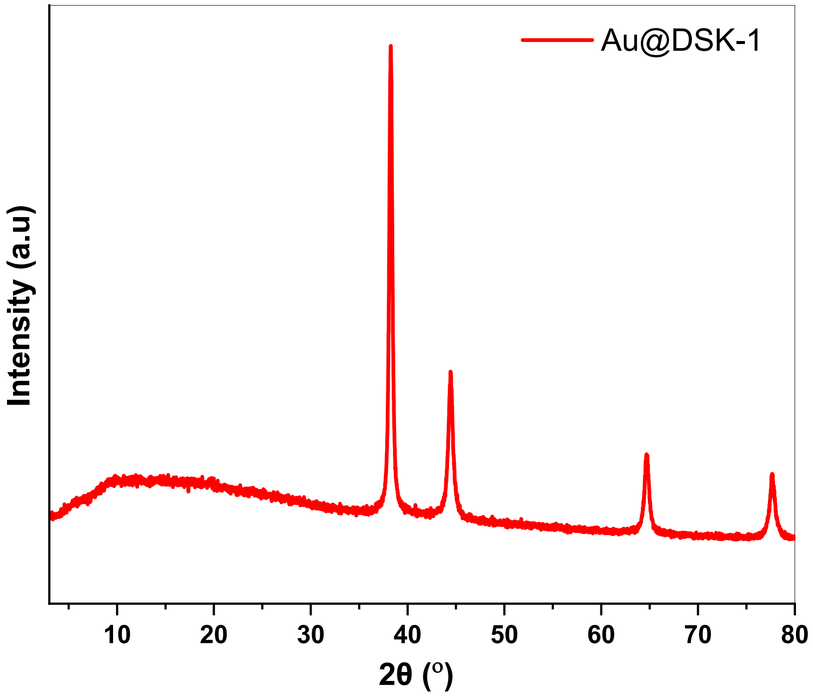

Figure S20. PXRD pattern of Au@DSK-1 shows the diffraction features of gold ions present in the sample matrix.

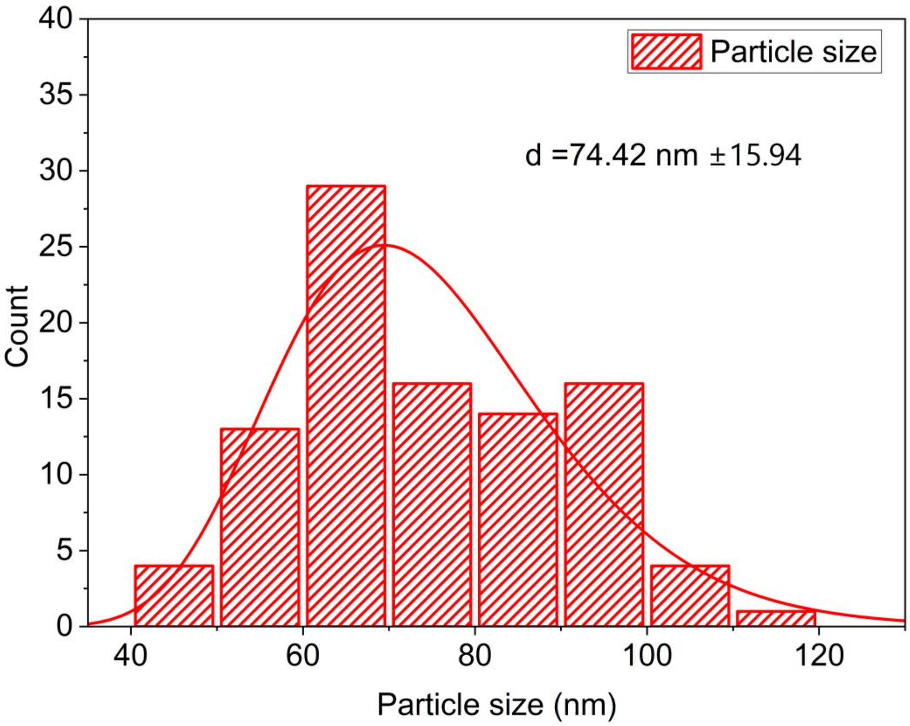

Figure S21. Particle size distribution curve of gold NPs obtained from TEM image of Au@DSK-1.

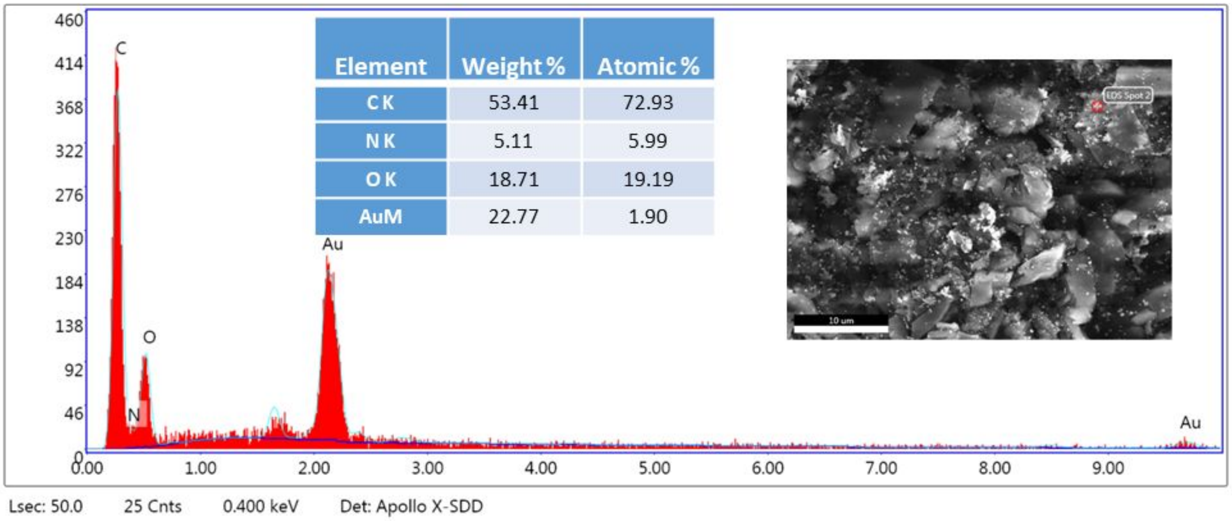

Figure S22. EDAX mapping analysis of Au@DSK-1 using SEM shows the presence of C, N, O, and Au constituents.

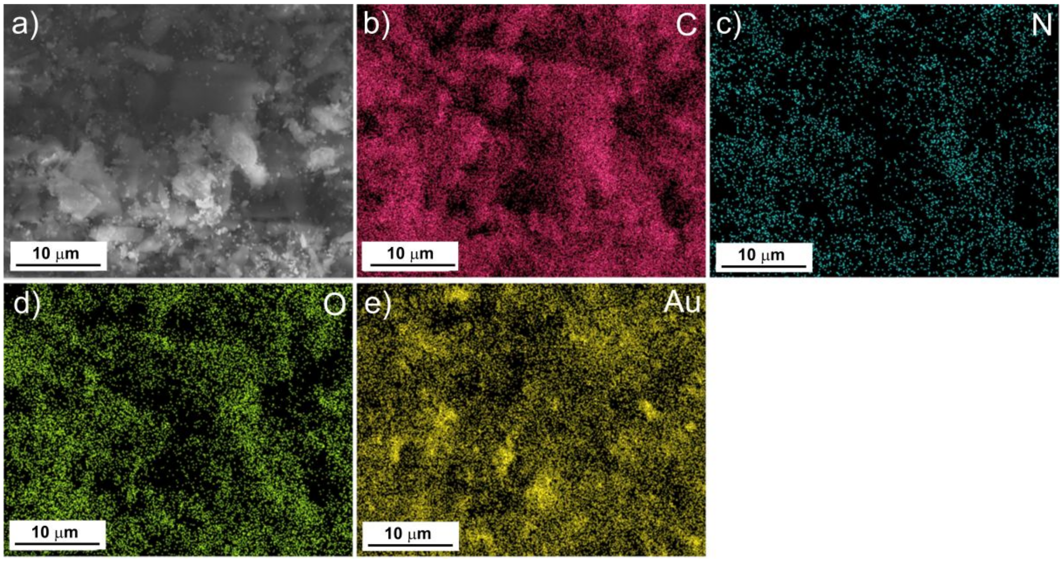

Figure S23. EDAX mapping performed using SEM for C, N, O, and Au elements present in Au@DSK-1.

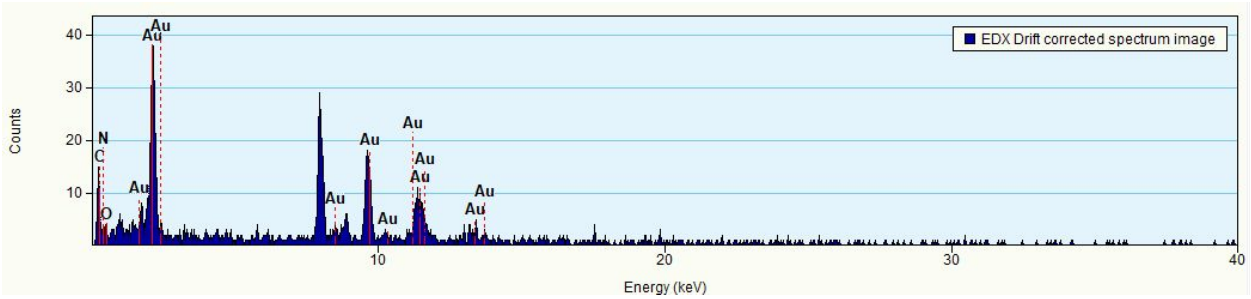

Figure S24. EDX mapping performed using TEM for C, N, O, and Au elements present in Au@DSK-1.

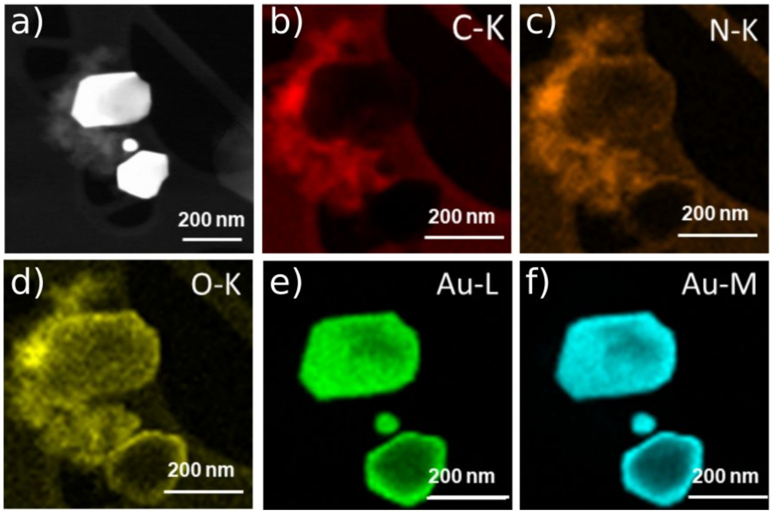

Figure S25. EDX mapping performed using TEM for C, N, O, and Au elements present in Au@DSK-1.

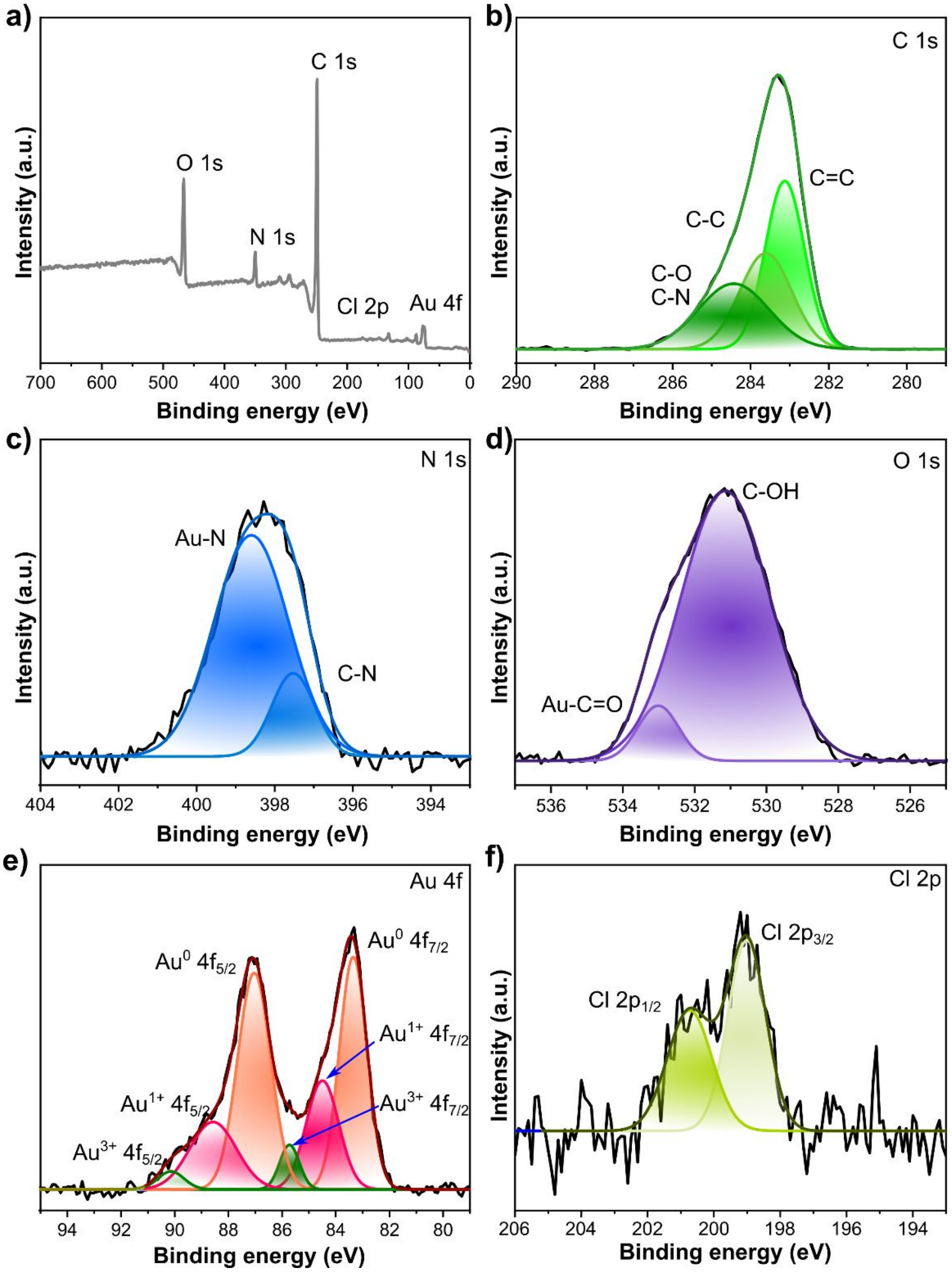

Figure S26. XPS profile of Au@DSK-1. A) full survey, b) C 1s, c) N 1s, d) O 1s, e) Au 4f, and f) Cl 2p.

Table S5. XPS profile data for pristine DSK-1 and Au@DSK-1.

	**DSK-1**		**Au@DSK-1**
C=C	282.98	C=C	283.11
C-C	283.60	C-C	283.60
C-O and C-N	284.40	C-O and C-N	284.42
C-N	397.92	Au-C-N	397.54
N-H	399.32	Au-N-H	398.61
C-OH	531.11	C-OH…Au	531.17
COOH	533.01	CH(O)…Au	533.04
		Au^{0} $4f_{7/2}$	83.33
		Au^{1+} $4f_{7/2}$	84.47
		Au^{3+} $4f_{7/2}$	85.70
		Au^{0} $4f_{5/2}$	87.01
		Au^{1+} $4f_{5/2}$	88.53
		$Au^{3+v}4f_{5/2}$	90.10
		Cl $2p_{3/2}$	199.00
		Cl $2p_{1/2}$	200.73

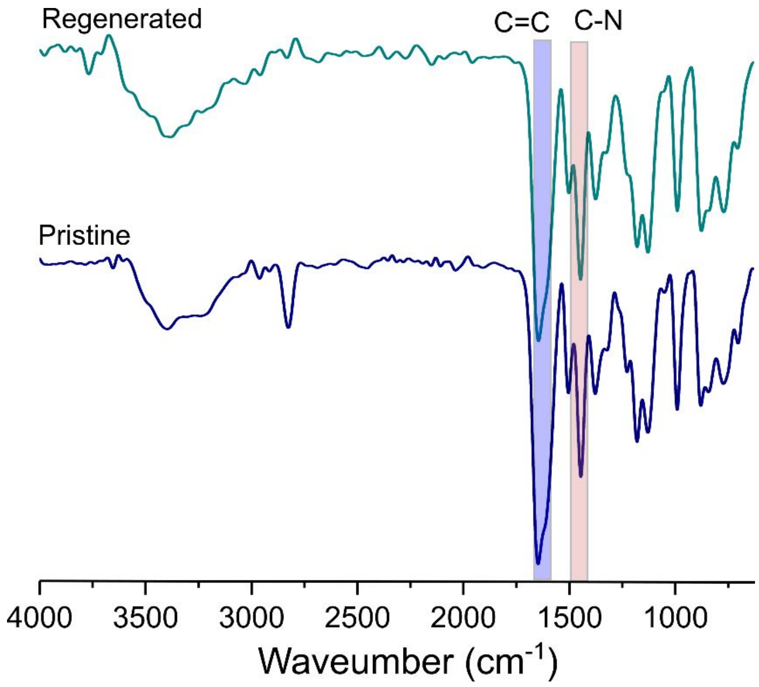

Figure S27. FTIR spectra of DSK-1 and after regeneration from e-waste solution.

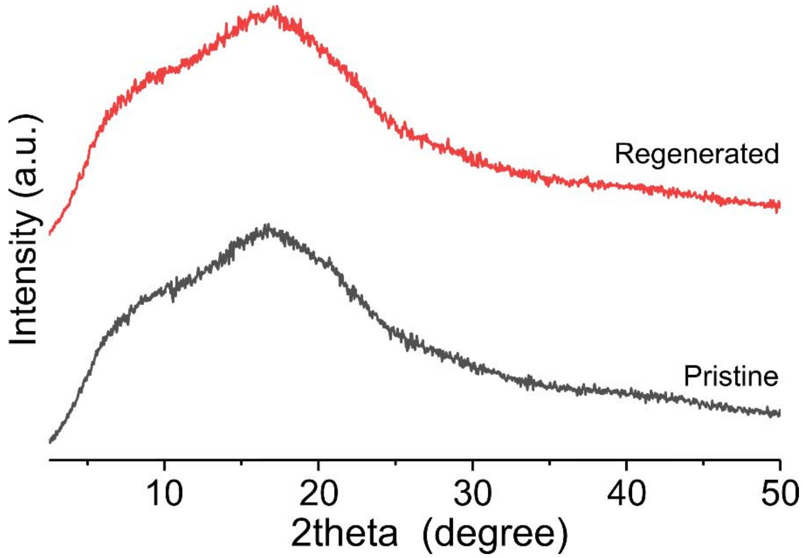

Figure S28. PXRD pattern of DSK-1 and after regeneration from e-waste solution.

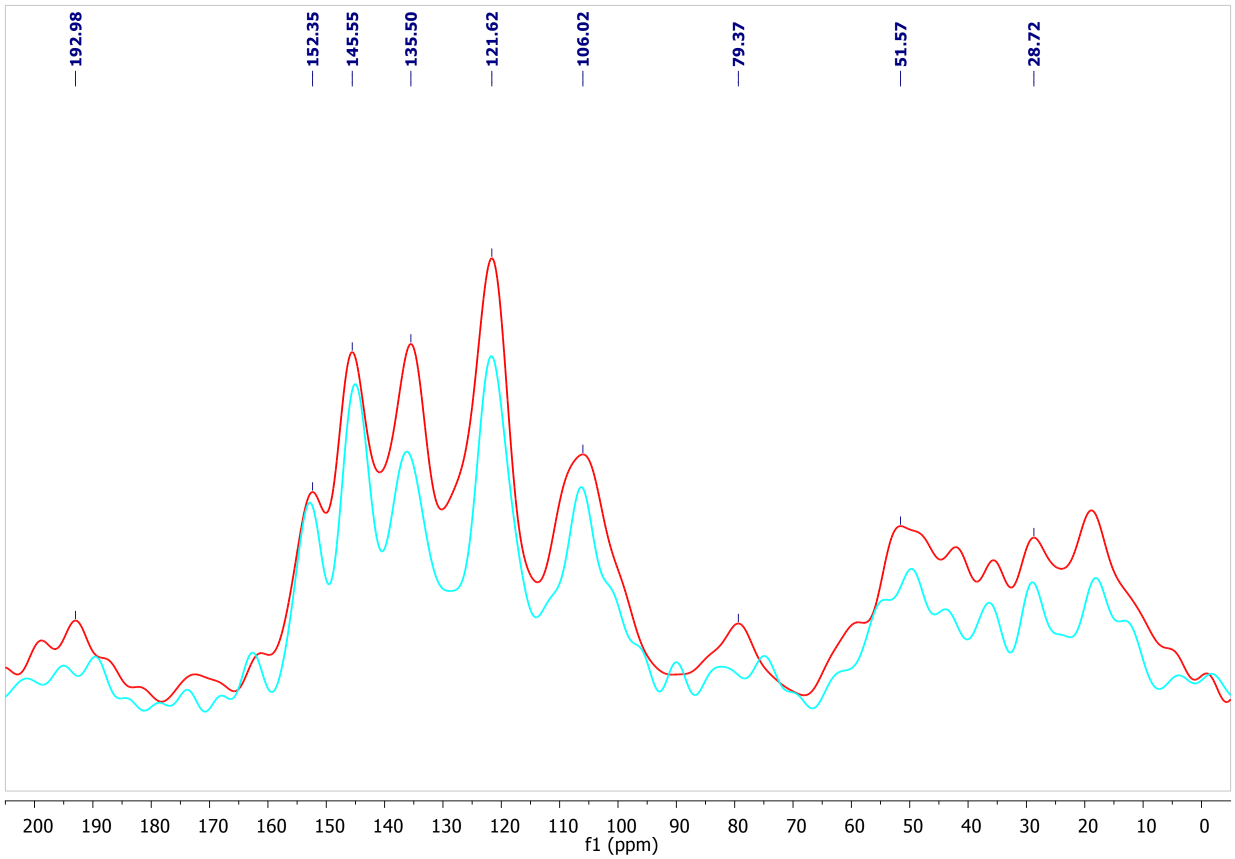

Figure S29. Comparison of solid-state NMR spectra of DSK-1 pristine (red) and after recovery from gold adsorption studies (blue) shows nearly negligible changes suggesting the integrity of the sample is well maintained.

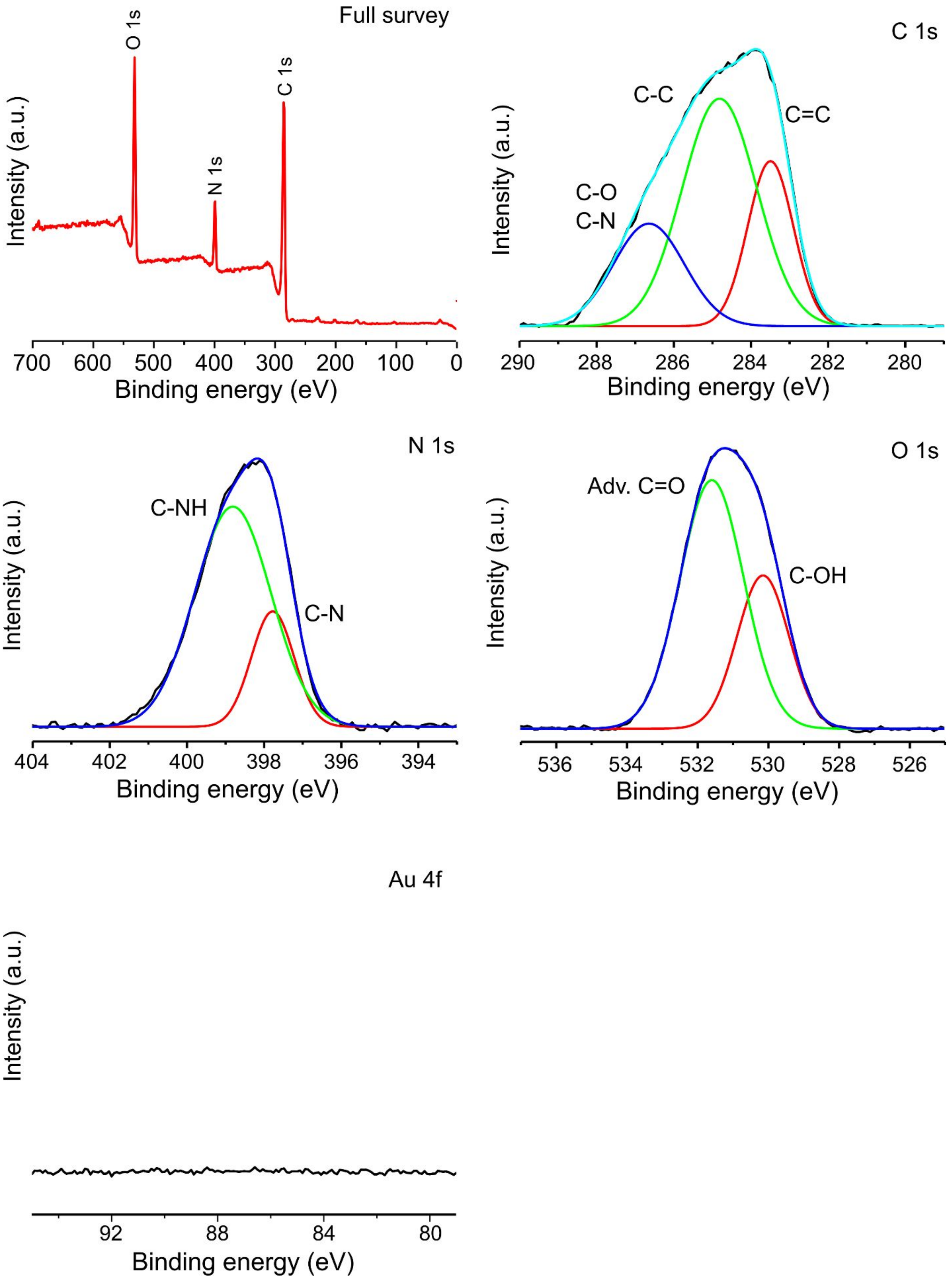
Full survey
O 1s
N 1s
C 1s
Intensity (a.u.)
700 600 500 400 300 200 100 0
Binding energy (eV)
C 1s
C-C
C=C
C-O
C-N
290 288 286 284 282 280
N 1s
C-NH
C-N
404 402 400 398 396 394
O 1s
Adv. C=O
C-OH
536 534 532 530 528 526
Au 4f
92 88 84 80

Figure S30. XPS profile of DSK-1 recovered after gold adsorption studies shows negligible changes in the C1s, N1s, and O 1s deconvoluted profiles suggesting the integrity of the samples is well maintained.

Table S6. Comparison of our synthesized CONs with reported porous materials for gold adsorption.

**Material**	**t (min)**	**Q_m (mg g^{-1})**	**No. of competing ions in real e-waste**	**Uptake in real e-waste (%)**	**Ref.**
DSK-1	2880	5560.31	12	99	This work
DSK-2	2880	1621.92	-	-	This work
T-PAC	0.5	2260	4	99	[1]
V-PPOP-Br	300	748.23	5	95.28	[2]
PAF-1-thiourea	5	2629.87	10	98.73	[3]
BIT-POP-14–BIT-POP-17	5040	2321	4	95	[4]
IPcomp-9	30	2349	3	99	[5]
FMOF-Co	10	1953.7	4	85	[6]
COF-HNU25	8	1725	2	99.9	[7]
im-PYTA-PZDH-COF	10	1558	4	99.95	[8]
Imi-PPOPs-Br	300	1543	5	89.02	[9]
Pc–POSS–POP	60	862.07	9	94.6	[10]
POPcarbene	360	2090	4	92.75	[11]
M−Cu−BDC−NH2	15	1184	10	85	[12]
PYTA-PZDH-COF	30	2314	7	100	[13]
BUT-33–PpPD	0.75	1600	4	99	[14]
TD-POP@CS/PEI-2	800	3344	5	76	[15]
COP-212	60	1250	13	95.6	[16]
N+-PYTA-PATA-COF	30	1834	4	93.87	[17]
TzDa-COF	30	1866	-	-	[18]
UKM-78	180	1480.04	-	-	[19]
Por-net	2880	1250	-	-	[20]
PYTA-BPDH-COF	30	1810	-	-	[13]
EDTA-TAB-PAN	30	2040	-	-	[21]
DB18C6-HCP	40	1667	-	-	[22]
PYTA-PATA-COF	30	1774	-	-	[17]
PYTA-BDTA-COF	30	1752	-	-	[17]
PYTA-TDTA-COF	30	1880	-	-	[17]
TTASDFP	2	245	-	-	[23]
TpTsc	2880	4400	-	-	[24]

1′@IL (Zr(IV)MOF)	1	1208.5	7	96	[25]
Cu-BDC-NH_2	30	1184	11	85	[26]
Ionic MOP/COF	16	1689	-	-	[27]

Table S7: DFT binding energies of gold species in DSK-1 and DSK-2

DSK-1	Au	-70.4
	$AuCl_4^-$	-118.0
DSK-2	Au	-137.9
	$AuCl_4^-$	-122.2

References

[1] J. Jiang, J. Kou, Q. Wu, L. Chen, Y. Geng, G. Shan, C. Sun, Z. Su, X. Wang, Anion-π Interactions on Functionalized Porous Aromatic Cages for Gold Recovery from Complex Aqueous with High Capacity, Angewandte Chemie International Edition 64 (2025) e202410665. https://doi.org/10.1002/anie.202410665.

[2] Y. Chen, Z. Li, R. Ding, T. Liu, H. Zhao, X. Zhang, Construction of porphyrin and viologen-linked cationic porous organic polymer for efficient and selective gold recovery, Journal of Hazardous Materials 426 (2022) 128073. https://doi.org/10.1016/j.jhazmat.2021.128073.

[3] T. Ma, R. Zhao, Z. Li, X. Jing, M. Faheem, J. Song, Y. Tian, X. Lv, Q. Shu, G. Zhu, Efficient Gold Recovery from E-Waste via a Chelate-Containing Porous Aromatic Framework, ACS Appl. Mater. Interfaces 12 (2020) 30474–30482. https://doi.org/10.1021/acsami.0c08352.

[4] J. Shi, S.-Q. Peng, B. Kuang, S. Wang, Y. Liu, J.-X. Zhou, X. Li, M.-H. Huang, Porous Polypyrrolidines for Highly Efficient Recovery of Precious Metals through Reductive Adsorption Mechanism, Advanced Materials 36 (2024) 2405731. https://doi.org/10.1002/adma.202405731.

[5] D. Majumder, S. Fajal, M.M. Shirolkar, A. Torris, Y. Banyla, K. Biswas, S. Rasaily, S.K. Ghosh, Nano-Springe Enriched Hierarchical Porous MOP/COF Hybrid Aerogel: Efficient Recovery of Gold from Electronic Waste, Angewandte Chemie International Edition n/a (2024) e202419830. https://doi.org/10.1002/anie.202419830.

[6] J. Liu, Z. Deng, H. Yu, L. Wang, Ferrocene-based metal-organic framework for highly efficient recovery of gold from WEEE, Chemical Engineering Journal 410 (2021) 128360. https://doi.org/10.1016/j.cej.2020.128360.

[7] J. Qiu, C. Xu, X. Xu, Y. Zhao, Y. Zhao, Y. Zhao, J. Wang, Porous Covalent Organic Framework Based Hydrogen-Bond Nanotrap for the Precise Recognition and Separation of Gold, Angewandte Chemie International Edition 62 (2023) e202300459. https://doi.org/10.1002/anie.202300459.

[8] X. Yang, D. Jiang, Y. Cheng, Y. Fu, X. Li, G. Liu, X. Ding, B.-H. Han, Q. Xu, G. Zeng, Imidazopyridinium-Linked Covalent Organic Frameworks for Efficient Gold Recovery, Small Methods n/a (2024) 2401792. https://doi.org/10.1002/smtd.202401792.

[9] R. Ding, J. Liu, T. Wang, X. Zhang, Bottom-up synthesis of cationic porphyrin-based porous organic polymers for highly efficient and selective recovery of gold, Chemical Engineering Journal 449 (2022) 137758. https://doi.org/10.1016/j.cej.2022.137758.

[10] R. Ding, Y. Chen, Y. Li, Y. Zhu, C. Song, X. Zhang, Highly Efficient and Selective Gold Recovery Based on Hypercross-Linking and Polyamine-Functionalized Porous Organic Polymers, ACS Appl. Mater. Interfaces 14 (2022) 11803–11812. https://doi.org/10.1021/acsami.1c22514.

[11] X. Li, Y.-L. Wang, J. Wen, L. Zheng, C. Qian, Z. Cheng, H. Zuo, M. Yu, J. Yuan, R. Li, W. Zhang, Y. Liao, Porous organic polycarbene nanotrap for efficient and selective gold stripping from electronic waste, Nature Communications 14 (2023) 263. https://doi.org/10.1038/s41467-023-35971-w.

[12] Y. Xiang, C.-Y. Cheng, M.-H. Liu, W.-C. Bai, Z.-X. Zang, L. Xu, Y. Yu, G.-J. Liu, Efficient recovery of gold using Macroporous Metal-Organic framework prepared by the “MOF in MOF” method, Separation and Purification Technology 335 (2024) 126131. https://doi.org/10.1016/j.seppur.2023.126131.

[13] X. Yang, D. Jiang, Y. Fu, X. Li, G. Liu, X. Ding, B.-H. Han, Q. Xu, G. Zeng, Synergistic Linker and Linkage of Covalent Organic Frameworks for Enhancing Gold Capture, Small 20 (2024) 2404192. https://doi.org/10.1002/smll.202404192.

[14] T. Xue, T. He, L. Peng, O.A. Syzgantseva, R. Li, C. Liu, D.T. Sun, G. Xu, R. Qiu, Y. Wang, S. Yang, J. Li, J.-R. Li, W.L. Queen, A customized MOF-polymer composite for rapid gold extraction from water matrices, Science Advances 9 (n.d.) eadg4923. https://doi.org/10.1126/sciadv.adg4923.

[15] S. Chen, R. Ding, B. Li, J. Lu, X. Zhang, A robust aerogel incorporated with phthalocyanine-based porous organic polymers for highly efficient gold extraction, Separation and Purification Technology 354 (2025) 129451. https://doi.org/10.1016/j.seppur.2024.129451.

[16] Y. Hong, V. Rozyyev, C.T. Yavuz, Alkyl-Linked Porphyrin Porous Polymers for Gas Capture and Precious Metal Adsorption, Small Science 1 (2021) 2000078. https://doi.org/10.1002/smsc.202000078.

[17] M. Liu, D. Jiang, Y. Fu, G. Zheng Chen, S. Bi, X. Ding, J. He, B.-H. Han, Q. Xu, G. Zeng, Modulating Skeletons of Covalent Organic Framework for High-Efficiency Gold Recovery, Angewandte Chemie International Edition 63 (2024) e202317015. https://doi.org/10.1002/anie.202317015.

[18] S. Zhong, Y. Wang, T. Bo, J. Lan, Z. Zhang, L. Sheng, J. Peng, L. Zhao, L. Yuan, M. Zhai, W. Shi, Efficient and selective gold recovery from e-waste by simple and easily synthesized covalent organic framework, Chemical Engineering Journal 455 (2023) 140523. https://doi.org/10.1016/j.cej.2022.140523.

[19] G. Hu, Z. Wang, C. Xia, X. Wang, H. He, Z. Nie, S. Wang, W. Li, Regulating the Interface Polarity Distribution of Zr-Based MOFs by Amino Acid-Like Ligand Functionalization Enables Efficient Recovery of Gold, ACS Appl. Mater. Interfaces 16 (2024) 42976–42985. https://doi.org/10.1021/acsami.4c08841.

[20] A. Preetam, A. Modak, S.N. Naik, K.K. Pant, V. Kumar, Realistic Approach for Recovering Gold from Waste Electronics by Thiourea Leaching and Adsorption Using a Covalent Porphyrin/Triphenylamine-Based Porous Polymer, ACS Appl. Polym. Mater. 6 (2024) 3676–3689. https://doi.org/10.1021/acsapm.3c02740.

[21] X.-Y. Bian, Y.-Z. Cheng, W. Ji, Y. Tao, B. Yuan, D. Jiang, B.-W. Yao, X.-M. Dou, D.-H. Yang, X. Ding, B.-H. Han, Solution-processed porous organic polymer for gold and platinum recovery, Separation and Purification Technology 353 (2025) 128177. https://doi.org/10.1016/j.seppur.2024.128177.

[22] H.-Y. Kong, T.-X. Wang, Y. Tao, X. Ding, B.-H. Han, Crown ether-based hypercrosslinked porous polymers for gold adsorption, Separation and Purification Technology 290 (2022) 120805. https://doi.org/10.1016/j.seppur.2022.120805.

[23] S. Abubakar, G. Das, T. Prakasam, A. Jrad, F. Gándara, S. Varghese, T. Delclos, M.A. Olson, A. Trabolsi, Enhanced Removal of Ultratrace Levels of Gold from Wastewater Using Sulfur-Rich Covalent Organic Frameworks, ACS Appl. Mater. Interfaces 17 (2025) 17794–17803. https://doi.org/10.1021/acsami.4c03685.

[24] L. Zhang, Q.-Q. Zheng, S.-J. Xiao, J.-Q. Chen, W. Jiang, W.-R. Cui, G.-P. Yang, R.-P. Liang, J.-D. Qiu, Covalent organic frameworks constructed by flexible alkyl amines for efficient gold recovery from leaching solution of e-waste, Chemical Engineering Journal 426 (2021) 131865. https://doi.org/10.1016/j.cej.2021.131865.

[25] S. Mukherjee, P. Mandal, A. N. Panda, S. Biswas, Ionic-Liquid-Tethered Cationic MOF for Adsorption of Gold from Aqueous Matrices and Electronic Waste. ACS Applied Nano Materials *9 (*2025) 511–521. https://doi.org/10.1021/acsanm.5c04758.

[26] Y. Xiang, C. Cheng, M. Liu, W. Bai, Z. Zang, L. Xu, Y. Yu, G. Liu, Efficient recovery of gold using Macroporous Metal-Organic framework prepared by the'MOF in MOF'method. Separation and Purification Technology 335 *(*2024) 126131. https://doi.org/10.1016/j.seppur.2023.126131.

[27] D. Majumder, S. Fajal, M. M. Shirolkar, A. Torris, Y. Banyla, K. Biswas, S. Rasaily, S. K. Ghosh, Nano-Springe Enriched Hierarchical Porous MOP/COF Hybrid Aerogel: Efficient Recovery of Gold from Electronic Waste. Angewandte Chemie International Edition 64 *(*2025) e202419830. https://doi.org/10.1002/anie.202419830.
